# SARS‐CoV‐2 N Protein Induces Acute Kidney Injury via Smad3‐Dependent G1 Cell Cycle Arrest Mechanism

**DOI:** 10.1002/advs.202103248

**Published:** 2021-11-23

**Authors:** Wenbiao Wang, Junzhe Chen, Dingwen Hu, Pan Pan, Liying Liang, Wenjing Wu, Ying Tang, Xiao R. Huang, Xueqing Yu, Jianguo Wu, Hui Y. Lan

**Affiliations:** ^1^ Departments of Medicine and Therapeutics Li Ka Shing Institute of Health Sciences, and Lui Che Woo Institute of Innovative Medicine The Chinese University of Hong Kong Hong Kong 999077 China; ^2^ Guangdong Provincial Key Laboratory of Virology Institute of Medical Microbiology Jinan University Guangzhou 510632 China; ^3^ Guangdong‐Hong Kong Joint Laboratory for Immunological and Genetic Kidney Disease Guangdong Academy of Medical Science Guangdong Provincial People's Hospital Guangzhou 510080 China; ^4^ The Chinese University of Hong Kong‐Guangdong Academy of Sciences/Guangdong Provincial People's Hospital Joint Research Laboratory on Immunological and Genetic Kidney Diseases The Chinese University of Hong Kong Hong Kong 999077 China; ^5^ Department of Nephrology The Third Affiliated Hospital Southern Medical University Guangzhou 510080 China; ^6^ State Key Laboratory of Virology College of Life Sciences Wuhan University Wuhan 430072 China

**Keywords:** acute kidney injury, G1 cell cycle, N protein, p21, SARS‐CoV‐2, Smad3, TGF‐*β*

## Abstract

COVID‐19 is infected by severe acute respiratory syndrome coronavirus 2 (SARS‐CoV‐2) and can cause severe multiple organ injury and death. Kidney is one of major target organs of COVID‐19 and acute kidney injury (AKI) is common in critically ill COVID‐19 patients. However, mechanisms through which COVID‐19 causes AKI remain largely unknown and treatment remains unspecific and ineffective. Here, the authors report that normal kidney‐specifically overexpressing SARS‐CoV‐2 N develops AKI, which worsens in mice under ischemic condition. Mechanistically, it is uncovered that SARS‐CoV‐2 N‐induced AKI is Smad3‐dependent as SARS‐CoV‐2 N protein can interact with Smad3 and enhance TGF‐*β*/Smad3 signaling to cause tubular epithelial cell death and AKI via the G1 cell cycle arrest mechanism. This is further confirmed in Smad3 knockout mice and cells in which deletion of Smad3 protects against SARS‐CoV‐2 N protein‐induced cell death and AKI in vivo and in vitro. Most significantly, it is also found that targeting Smad3 with a Smad3 pharmacological inhibitor is able to inhibit SARS‐CoV‐2 N‐induced AKI. In conclusion, the authors identify that SARS‐CoV‐2 N protein is a key mediator for AKI and induces AKI via the Smad3‐dependent G1 cell cycle arrest mechanism. Targeting Smad3 may represent as a novel therapy for COVID‐19‐asscoaited AKI.

## Graphical abstract

In this article, the authors discover that severe acute respiratory syndrome coronavirus 2 (SARS‐CoV‐2) N protein can bind and activate Smad3 to induce kidney cell death and cause acute kidney injury (AKI) via p21‐dependent G1 cell cycle arrest mechanism. Targeting Smad3 with a pharmacological inhibitor SIS3 can inhibit SARS‐CoV‐2 N‐induced AKI.

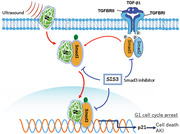

## 1 Introduction

COVID‐19 is caused by severe acute respiratory syndrome coronavirus 2 (SARS‐CoV‐2) and has resulted in hundreds of millions confirmed cases and more than 4 million deaths worldwide. SARS‐CoV‐2 virion contains four major structural proteins: spike (S), envelope (E), membrane (M), and nucleocapsid (N).^[^ ^1^, ^2^ ^]^ Of them, the S protein has been well studied and has resulted in clinical diagnosis and vaccination worldwide. Although it is well recognized that the S protein plays a critical role in COVID‐19 infection by mediating the SARS‐CoV‐2 entry into the host cells through the angiotensin‐converting enzyme 2 (ACE2),^[^ ^3^ ^]^ it remains unclear which viral proteins can cause cell injury as the consequences of COVID‐19 infection. It has been reported that the N protein can bind to the RNA genome after SARS coronaviruses (SARS‐CoV) or SARS‐CoV‐2 entering cells and plays an essential role in viral RNA transcription and replication,^[^ ^4^ ^]^ type I interferon signaling pathway,^[^ ^5^, ^6^ ^]^ activation of NLRP3 inflammation,^[^ ^7^ ^]^ activation of TGF‐*β*/Smad3 signaling,^[^ ^8^ ^]^ and cell cycle progression and apoptosis.^[^ ^9^, ^10^ ^]^ Thus, studying the intracellular mechanisms of SARS‐CoV‐2 N protein may provide a better understanding of the pathophysiological process in response to COVID‐19 infection.

Clinically, most COVID‐19 patients have mild symptoms, but they can develop into severe conditions with acute respiratory distress syndrome (ARDS), multiple organ failure, and even death in patients with underlying diseases or stress conditions.^[^ ^11^ ^]^ Single‐cell RNA sequencing analysis reveals that ACE2 is mainly expressed by proximal tubular cells and glomerular parietal epithelial cells.^[^ ^12^ ^]^ It has also been reported that SARS‐CoV‐2 can directly infect human kidney organoids via the ACE2 receptor.^[^ ^13^ ^]^ Acute kidney injury (AKI) is common in critically ill COVID‐19 patients, characterized by elevated serum levels of creatinine, tubular necrosis, and renal inflammation.^[^ ^14^ ^]^ In COVID‐19 patients died from AKI, expression of SARS‐CoV‐2 S and N proteins are detectable in the renal tubular epithelial cells (TECs),^[^ ^14^, ^15^, ^16^, ^17^ ^]^ providing direct evidence for SARS‐CoV‐2‐associated AKI. Epidemically, the incidence of AKI in COVID‐19 patients is variable and depends upon the severity of COVID‐19 infection with or without underlying stress and disease conditions, ranging from 10.5% in the early report in Wuhan,^[^ ^18^ ^]^ to the much higher rate with 23–37% in critically ill hospitalized patients in the New York city.^[^ ^19^, ^20^, ^21^ ^]^ Thus, AKI is the second highest complication in critically ill COVID‐19 patients after the acute respiratory failure.^[^ ^22^, ^23^ ^]^ However, mechanisms of SARS‐CoV‐2‐induced AKI remain largely unknown. Although it is now well understood that the S protein can function to mediate the SARS‐CoV‐2 entry into the host cells via its cellular receptor ACE2,^[^ ^3^ ^]^ mechanisms of SARS‐CoV‐2‐induced cell death in AKI after COVID‐19 infection remain largely unexplored and treatment remains unspecific and ineffective. Thus, identifying and uncovering mechanisms specifically related to a SARS‐CoV‐2 protein that can induce cell death in AKI after the COVID‐19 infection is urgently needed and is the first step toward the development of novel therapy for COVID‐19‐associated AKI.

It is well recognized that TEC death is a major pathological feature of AKI and cell cycle arrest determines the disease progression or regression after AKI,^[^ ^24^ ^]^ which is also influenced by many stress factors and the underlying diseases.^[^ ^25^, ^26^ ^]^ It is well known that TGF‐*β* can cause cell death by arresting cell cycle at the G1 phase via the Smad‐dependent mechanism.^[^ ^27^, ^28^ ^]^ We have also shown that TGF‐*β* signaling plays a critical role in AKI as mice lacking TGF‐*β* receptor II or Smad3 are protected from ischemic and cisplatin‐induced AKI.^[^ ^29^, ^30^ ^]^ Our recent studies also detected that Smad3 mediates AKI by directly binding to cyclin‐dependent kinase inhibitor proteins p21/p27 to cause TEC death via the G1 cell cycle arrest, which is promoted by ischemic and high inflammatory status with CRP conditions but blocked by genetically deleting Smad3 gene or by pharmacological inhibition of Smad3 with a Smad3 inhibitor SIS3.^[^ ^30^, ^31^, ^32^, ^33^ ^]^ Results from these studies reveal that activation of TGF‐*β*/Smad3 signaling plays a critical role in the development of AKI. However, the role of Smad3 in COVID‐19‐associated AKI remains unknown. As SARS‐CoV N protein is able to interact with Smad3 and activate TGF‐*β*/Smad3 signaling to promote fibrosis response,^[^ ^8^ ^]^ we thus hypothesized that SARS‐CoV‐2 N protein may be able to trigger Smad3 signaling to induce TEC death and AKI via the Smad3‐dependent G1 cell cycle arrest mechanism, and targeting Smad3 may be a novel and specific therapy for SARS‐CoV‐2‐associated AKI. In the present study, we examined this hypothesis by first determining whether SARS‐CoV‐2 N protein can directly induce AKI by kidney‐specifically overexpressing SARS‐CoV‐2 N protein in normal mice using our well‐established noninvasive ultrasound‐microbubble technique. Because severe AKI is developed in critically ill COVID‐19 patients with ischemic and inflammatory stress conditions clinically,^[^ ^34^ ^]^ we then determined if overexpression of renal SARS‐CoV‐2 N protein can exacerbate the severity of AKI in a mouse model of ischemic reperfusion injury (IRI). As the SARS‐CoV N protein can directly interact with Smad3,^[^ ^8^ ^]^ we also investigated the signaling mechanism through which SARS‐CoV‐2 N induces AKI in Smad3 knockout (KO) mice and cells in vivo and in vitro. Finally, the therapeutic potential for SARS‐CoV‐2 N‐induced AKI was developed by targeting Smad3 with a Smad3 inhibitor SIS3.

## 2 Results

### 2.1 SARS‐CoV‐2 N Protein Can Induce AKI and Causes Severe AKI under Ischemic Stress Conditions

As SARS‐CoV‐2 N protein expression is detectable in renal TECs of AKI patients died from SARS‐CoV‐2 infection,^[^ ^14^, ^15^, ^16^, ^17^ ^]^ we first determined whether SARS‐CoV‐2 N protein can directly induce AKI by kidney‐specifically transfecting the SARS‐CoV‐2‐N protein‐expressing plasmid into the normal mouse kidneys using our well‐established noninvasive ultrasound‐microbubble technique as previously described.^[^ ^35^, ^36^ ^]^ We found that kidney‐specific transfecting SARS‐CoV‐2 N‐expressing plasmids resulted in a dose‐dependently overexpressing the SARS‐CoV2 N protein in the mouse kidneys, with an optimal dose at 200 µg/mouse (200‐N) on day 3, which was demonstrated by a strong intracellular granular‐expressing pattern of SARS‐CoV‐2 N protein in more than 80% of kidney cells, primarily in TECs and to a less extent in glomerular cells and vascular cells (Figure S1A,C, Supporting Information). Unexpectedly, overexpressing SARS‐CoV‐2‐N protein in the kidney resulted in the development of AKI as evidenced by causing tubular necrosis (Figure S1B,D, Supporting Information) and an increase in serum creatinine (Figure S2A, Supporting Information) in a dose‐dependent manner, peaking at 200 µg/mouse. All these findings provided a direct evidence for a pathogenic role of SARS‐CoV‐2 N in the induction of AKI. It should be known that the use of ultrasound‐microbubble‐mediated kidney‐specifically transfecting SARS‐CoV‐2 N protein‐expressing plasmids did not cause the systemic toxicity and other organs' injury as SARS‐CoV‐2 N protein expression was not detectable in the liver and heart tissues with normal serum levels of alanine aminotransferase (ALT), aspartate aminotransferase (AST), and lactate dehydrogenase (LDH) (Figure S2B–E, Supporting Information).

We then determined the time‐dependent injury of AKI induced by kidney specifically overexpressing the optimal dose of SARS‐CoV‐2 N expressing plasmids (200 µg/mouse, 200‐N) in normal mouse kidneys over days 1, 2, 3, 4, and 5 after ultrasound‐microbubble‐mediated SARS‐CoV‐2 N gene transfer. Immunohistochemistry showed that compared to empty vector controls (VC), SARS‐CoV‐2‐N protein was highly expressed by TECs, glomerular cells, and vascular cells in a time‐dependent manner, peaking at day 2 and then decreasing over the 3–5‐day period (Figure S3A,C, Supporting Information). This was associated with the development of AKI by a significant increase in acute tubular necrosis and serum levels of creatinine at day 1, peaking at day 2 and then decreasing over the next 3 days to become insignificant on day 5 (Figure S3B,D,E, Supporting Information). Based on the results obtained from both dose‐ and time‐dependent studies, SARS‐CoV‐2 N expressing plasmids at the dose of 200 µg/mouse was selected as an optimal dosage and day 2 after the N gene transfection was used as the peak time of AKI for examining the pathogenic role of SARS‐CoV‐2 N in the development of AKI under normal and ischemic stress conditions.

**Figure 1:**
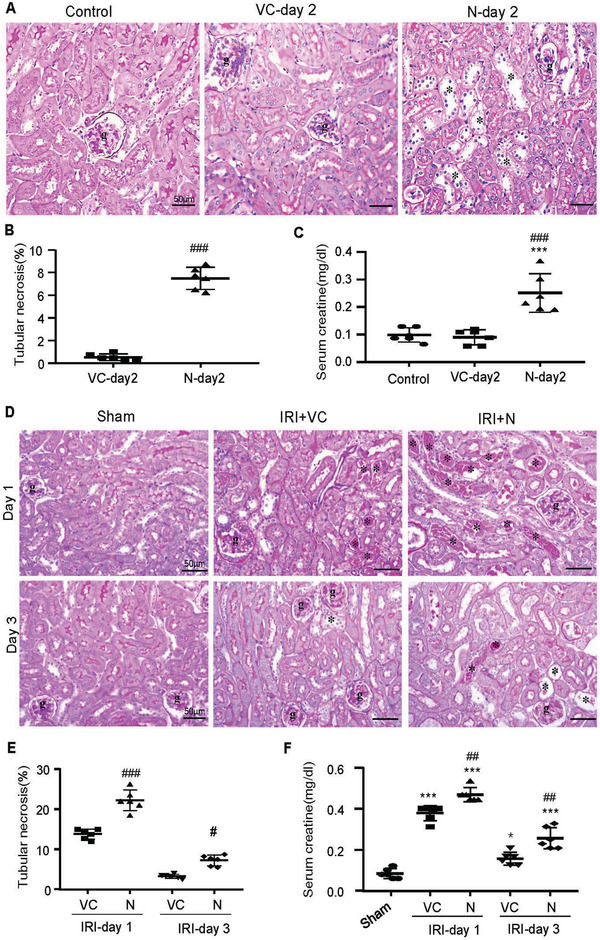
Ultrasound‐microbubble‐mediated kidney‐specifically overexpressing SARS‐CoV‐2 N protein induces AKI in normal mice, which becomes worse in mice with ischemic reperfusion injury (IRI). A–C) SARS‐CoV‐2 N induces AKI in normal mice. D–F) SARS‐CoV‐2 N causes severe AKI in IRI‐mice. A,D) PAS staining. B,E) Semiquantitative analysis of tubular necrosis. C,F) Serum levels of creatinine. Note that kidney‐specifically SARS‐CoV‐2 N‐expressing plasmid transfer (200 µg/per mice) causes moderate AKI in normal mice by significantly increasing tubular necrosis (*) and serum levels of creatinine, which becomes worse in IRI‐mice. Each dot represents one mouse and data are expressed as the mean ± SEM for groups of six mice. *p < 0.05, ***p < 0.001 versus sham or normal control group; #*p* < 0.05, ###*p* < 0.001 versus empty vector control (VC) group. g, glomerulus; scale bars = 50 µm.

Next, we examined the direct role of SARS‐CoV‐2 N in the pathogenesis of AKI by transfecting SARS‐CoV‐2 N‐expressing plasmid (200 µg/mouse) into the mouse kidney of normal mice and examined the AKI parameters on day 2 after the N gene transfer. Compared to the empty VC (VC‐Day2), kidney‐specifically overexpressing SARS‐CoV‐2 N protein resulted in the development of moderate tubular necrosis (**Figure** **1**A,B) and a significant increase in serum levels of creatinine (Figure 1C), revealing a direct role of SARS‐CoV‐2 N in AKI. To provide further evidence for a promoter role of SARS‐CoV‐2‐N protein in AKI under ischemic conditions that are mimicking critically ill COVID‐19 patients clinically, AKI was induced in mice 2 days after SARS‐CoV‐2‐N gene transfer by a well‐established IRI technique in which renal arteries were bilaterally clamped with the vascular clip for 30 min. Strikingly, compared to the control empty vector‐treated IRI‐mice, overexpression of SARS‐CoV‐2 N protein significantly enhanced IRI‐induced AKI, showing a massive tubular necrosis (Figure 1D,E) and higher levels of serum creatinine (Figure 1F) at day 1 after IRI. Moreover, mice overexpressing SARS‐CoV‐2 N protein also delayed the recovery time from IRI‐induced AKI as the degree of tubular necrosis and elevated serum levels of creatine remained significantly higher than the VC‐treated IRI mice on day 3 (Figure 1D–F). Taken together, these novel observations demonstrated that kidney‐specifically overexpressing SARS‐CoV‐2 N was able to directly induce AKI under normal conditions and was able to largely promote the severity of AKI under ischemic stress conditions. These findings are mimicking the clinical observations that AKI is less severe and uncommon in the healthy population with COVID‐19 infection but becomes worse in those with ischemic and inflammatory stress conditions.^[^ ^34^ ^]^

### 2.2 SARS‐CoV‐2 N Protein‐Induced AKI Is Associated with the Activation of Smad3‐p21‐G1 Cell Cycle Arrest Pathway

**Figure 2:**
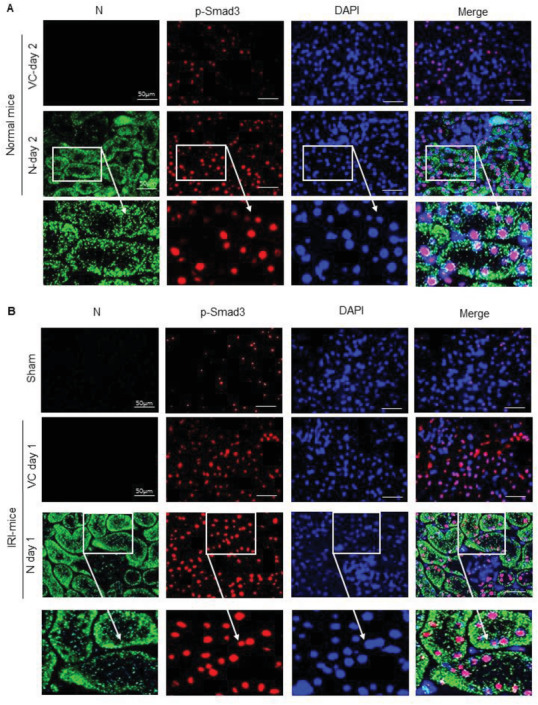
Tow‐color immunofluorescence detects that SARS‐CoV‐2 N protein is highly expressed by TECs and is colocalizing with activated Smad3 (p‐Smad3) in nuclei of TECs. A) SARS‐CoV‐2 N expression and activation of Smad3 signaling in normal mice. B) SARS‐CoV‐2 N expression and activation of Smad3 signaling in IRI‐mice. Note that SARS‐CoV‐2 N protein is highly expressed by TECs in a granular pattern and is colocalized with p‐Smad3 in nuclei of TECs. DAPI (blue, nuclei), p‐Smad3 (red), SARS‐CoV‐2 N protein (green). Scale bar = 50 µm.

**Figure 3:**
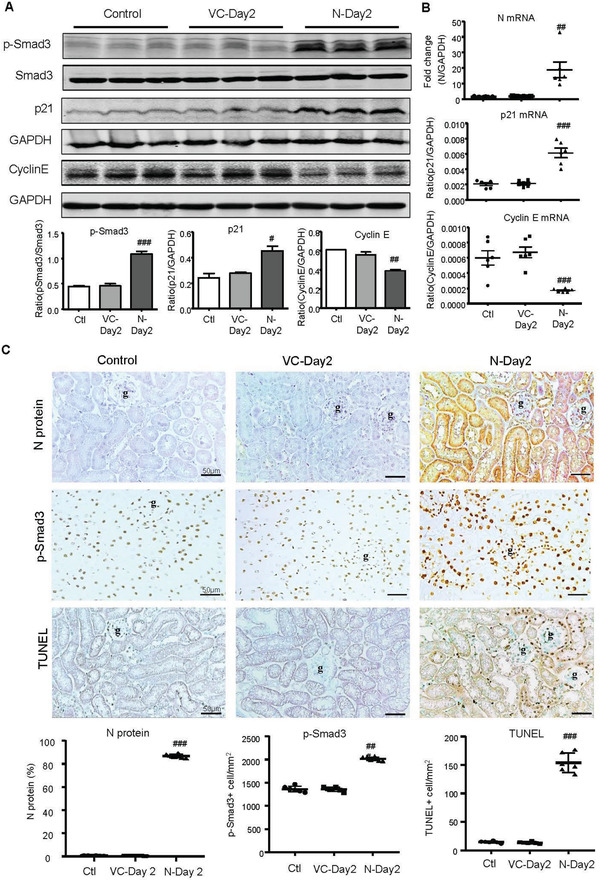
SARS‐CoV‐2 N protein‐induced AKI is associated with activation of Smad3‐p21‐dependent G1 cell cycle arrest pathway. A) Western blotting for activation of Smad3 (p‐Smad3) and expression of p21 and cyclin E. B) Real‐time PCR for levels of SARS‐CoV‐2 N, p21, and cyclin E mRNA expression. C) Immunohistochemistry for detecting SARS‐CoV‐2 N protein expression, activation of Smad3 (p‐Smad3 nuclear translocation), and cell apoptosis by TUNEL‐labeling. Note that overexpressing SARS‐CoV‐2 N in the normal mouse kidney resulted in a marked activation of Smad3, upregulation of p21 while inhibiting cyclin E, and a massive cell apoptosis. Each dot represents one mouse and data are expressed as the mean ± SEM for groups of six mice. #*p* < 0.05, ##*p* < 0.01, ###*p* < 0.001 versus empty vector control (VC‐Day2) group. g, glomerulus; scale bars = 50 µm.

**Figure 4:**
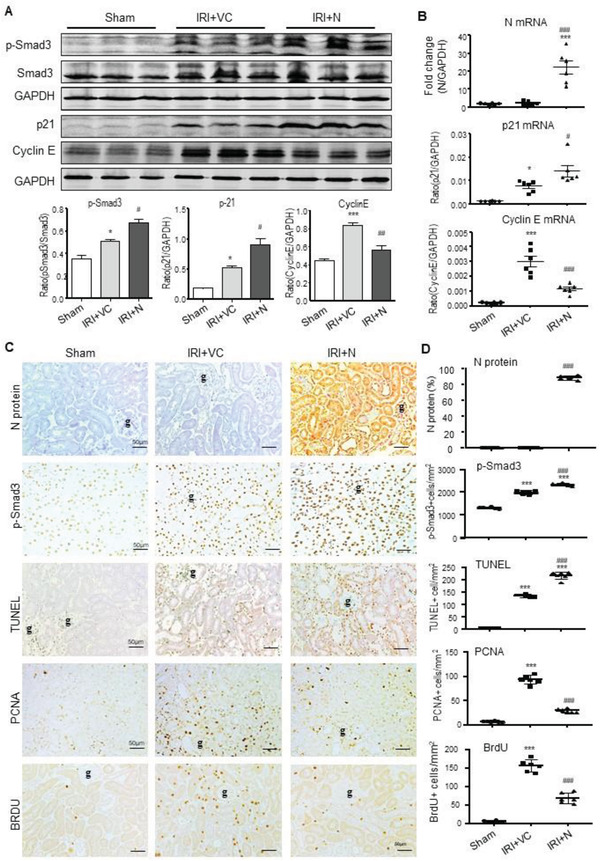
AKI‐induced by SARS‐CoV‐2 N protein is associated with activation of Smad3‐p21‐dependent G1 cell cycle arrest pathway under ischemic stress conditions. A) Western blotting for activation of Smad3 (p‐Smad3) and expression of p21 and cyclin E. B) Real‐time PCR for levels of SARS‐CoV‐2 N, p21, and cyclin E mRNA expression. C and D) Immunohistochemistry for detecting SARS‐CoV‐2 N protein expression, activation of Smad3 (p‐Smad3 nuclear translocation), cell apoptosis by TUNEL‐labeling, and TEC proliferation by PCNA‐labeling and S‐phase cell cycle by BrdU‐labeling. Note that overexpression of SARS‐CoV‐2 N protein can promote ischemic stress‐induced activation of Smad3 signaling to cause cell death via p21‐dependent G1 cell cycle arrest mechanism. Each dot represents one mouse and data are expressed as the mean ± SEM for groups of six mice. *p < 0.05, ***p < 0.001 versus sham group; #*p* < 0.05, ##*p* < 0.01, ###*p* < 0.001 versus AKI with empty vector control (IRI+VC) group. g, glomerulus; scale bars = 50 µm.

Our previous study has demonstrated that Smad3 can cause TEC death and AKI via the G1 cell cycle arrest mechanism by directly interacting with the cyclin‐dependent kinase inhibitors (p21/p27).^[^ ^30^, ^33^ ^]^ Here we examined whether AKI induced by kidney‐specifically overexpressing SARS‐CoV‐2 N protein is associated with activation of Smad3‐p21‐dependent G1 cell cycle arrest pathway in both normal and ischemic stress conditions. Two‐color immunofluorescence clearly detected that ultrasound‐microbubble‐mediated SARS‐CoV‐2 N gene transfer resulted in a strong N protein expression in both normal and IRI‐induced ischemic kidneys, presumably by TECs in a granular pattern, which was associated with a marked activation of Smad3 (p‐Smad3) with a tight colocalization of SARS‐CoV‐2 N protein and p‐Smad3 in the nuclei in both normal and ischemic kidneys (**Figure** **2**A,B). These observations suggest that overexpression of SARS‐CoV‐2 N protein in the kidney may trigger activation of Smad3 by enhancing Smad3 phosphorylation and p‐Smad3 nuclear translocation during the development of tubular necrosis. Further studies by western blotting, real‐time PCR, immunohistochemistry, and terminal deoxynucleotidyl transferase‐mediated dUTP nick end labeling (TUNEL) revealed that overexpression of SARS‐CoV‐2 N mRNA and protein was associated with a marked activation of Smad3 signaling and the development of G1 cell cycle arrest as detected by upregulating p21 while suppressing cyclin E in both mRNA and protein levels, resulting in massive TEC apoptosis as labeled by TUNEL‐positive cells (**Figure** **3**A–C). Similar results were also found in IRI‐induced AKI mice in which overexpression of SARS‐CoV‐2 N mRNA and protein largely promoted Smad3 signaling and TEC death (Figure 1D,E). This was also associated with a marked increase in p21‐dependent G1 cell cycle arrest (**Figure** **4**A,B), TEC apoptosis with many TUNEL‐positive TECs, and inhibition of TEC proliferation (PCNA+) and the entering into the S‐phase cell cycle with few BrdU+ TECs (Figure 4C,D). Taken together, our results revealed that overexpression of SARS‐CoV‐2 N protein may trigger activation of Smad3 signaling to cause cell death via the p21‐dependent G1 cell cycle arrest mechanism.

### 2.3 SARS‐CoV‐2 N Protein Induces AKI via a Smad3‐Dependent Mechanism

**Figure 5:**
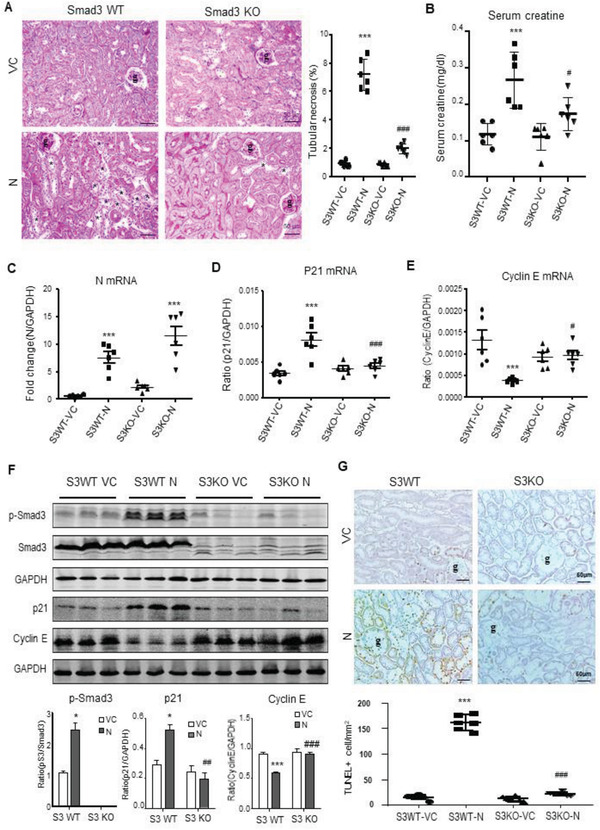
SARS‐CoV‐2 N protein induces AKI via a Smad3‐dependent mechanism. A) PAS staining and semiquantitative analysis of tubular necrosis. B) Serum creatinine. C–E) Real‐time PCR for levels of SARS‐CoV‐2 N, p21, and cyclin E mRNA expression. F) Western blotting for activation of Smad3 signaling (p‐Smad3) and expression of p21 and cyclin E. G) Immunohistochemistry for detecting cell apoptosis by TUNEL‐labeling. Note that mice lacking Smad3 are protected against SARS‐CoV‐2 N‐induced AKI. Each dot represents one mouse and data are expressed as the mean ± SEM for groups of six mice. *p < 0.05, ***p < 0.001 versus Smad3 WT with empty vector control (S3WT‐VC) group; #*p* < 0.05, ##*p* < 0.01, ###*p* < 0.001 versus Smad3 WT with SARS‐CoV‐2 N plasmid (S3WT‐N) group. g, glomerulus; scale bars = 50 µm.

We next examined the regulatory role of Smad3 in SARS‐CoV‐2 N protein‐induced AKI by ultrasound‐microbubble‐mediated kidney‐specifically transfecting the SARS‐CoV‐2 N protein expressing plasmids (200 µg/mouse) into the Smad3 wild‐type (WT) or KO mice. As shown in **Figure** **5**, periodic acid–Schiff (PAS)‐staining, real‐time PCR, western blotting, and TUNEL labeling detected that overexpression of SARS‐CoV‐2 N in Smad3 WT mouse kidneys caused moderate to severe AKI at day 2 by a significant increase in tubular necrosis and serum levels of creatinine (Figure 5A,B). This was associated with activation of Smad3‐p21 signaling while suppressing cyclin E to cause TEC death as detected by massive TUNEL‐positive cells (Figure 5C–G). In contrast, mice lacking Smad3 were protected against SARS‐CoV‐2 N protein‐induced AKI by blocking tubular necrosis (Figure 5A) and preventing an increase in serum creatinine (Figure 5B). This protective effect on SARS‐CoV‐2 N‐induced AKI in Smad3 KO mice was associated with inhibition of G1 cell cycle arrest by suppressing p21 while increasing cyclin E, thereby protecting TECs from apoptosis (Figure 5C–G). Interestingly, compared to Smad3 WT mice, mice lacking Smad3 showed a trend increase in renal SARS‐CoV‐2 N mRNA expression although AKI was prevented (Figure 5C), confirming an essential role for Smad3 in SARS‐CoV‐2 N‐induced AKI.

### 2.4 SARS‐CoV‐2 N Protein Induces TEC Arrest at the G1 Cell Cycle via a Smad3‐Dependent Mechanism In Vitro

**Figure 6:**
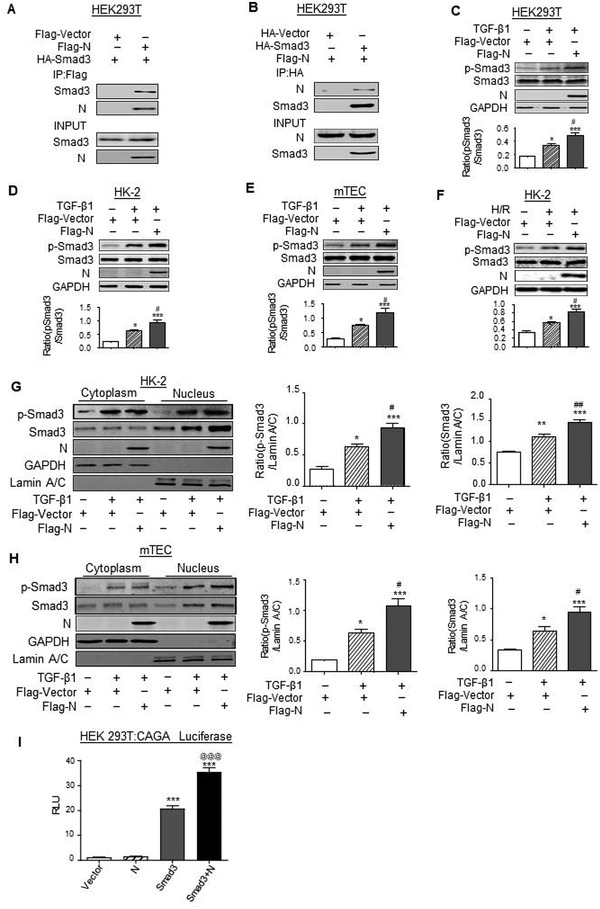
SARS‐CoV‐2 N protein can directly interact with Smad3 and promotes Smad3 signaling upon TGF‐*β*1 or H/R injury stimulation in vitro. A,B) The interaction between Flag‐N and HA‐Smad3 by Co‐IP in HEK293T cells co‐expressing Flag‐SARS‐CoV‐2 N and HA‐Smad3. C–F) Western blotting for activation of Smad3 (p‐Smad3) and expression of SARS‐CoV‐2 N protein in response to TGF‐*β* (5 ng mL^−1^ for 30 min) by HEK293T (C), human tubular epithelial cells (HK‐2) (D), mouse tubular epithelial cells (mTECs) (E), and in response to H/R injury by HK‐2 (F). G,H) Western blotting for activation of Smad3 (nuclear p‐Smad3) and SARS‐CoV‐2 N expression in cytoplasmic and nuclear protein fractions in response to TGF‐*β*1 (5 ng mL^−1^ for 30 min) by HK‐2 (G) and mTECs (H). I) HEK293T cells were cotransfected with CAGA‐luciferase reporter, HA‐Smad3, and Flag‐N. Overexpression of SARS‐CoV‐2 N protein enhanced Smad3 promoter luciferase activities in HEK293T. Data was expressed as means ± SEM for at least three independent experiments. *p < 0.05, **p < 0.01, ***p < 0.001 versus Flag‐Vector; #*p* < 0.05, ##*p* < 0.01, ###*p* < 0.001 versus Flag‐Vector with TGF‐*β*1 or H/R.

**Figure 7:**
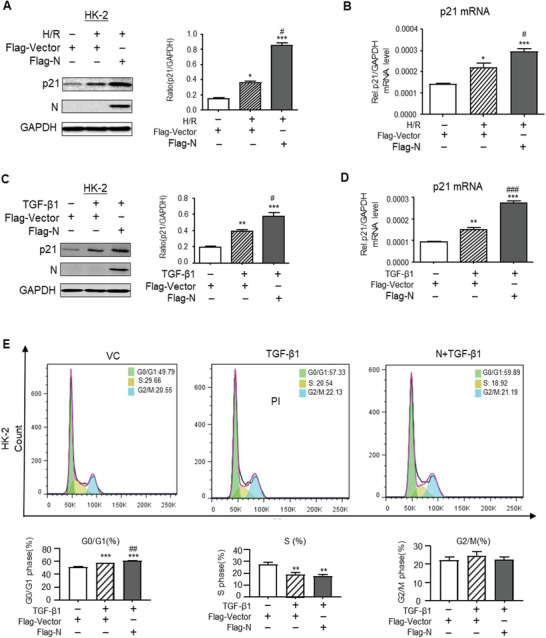
SARS‐CoV‐2 N protein promotes the expression of p21 and cell cycle arrest in vitro. A,B) Western blotting and real‐time PCR for expression of p21 in response to overexpressing SARS‐CoV‐2 N protein and in response to H/R injury. C,D) Western blotting and real‐time PCR for expression of p21 in response to overexpressing SARS‐CoV‐2 N protein and addition of TGF‐*β* (5 ng mL^−1^). E) Cell cycle progression in response to overexpressing SARS‐CoV‐2 N protein and addition of TGF‐*β* (5 ng mL^−1^) by flow‐cytometry analysis. Data was expressed as means ± SEM for at least three independent experiments. Data was expressed as means ± SEM for at least three independent experiments. *p < 0.05, **p < 0.01, ***p < 0.001 versus Flag‐Vector; #*p* < 0.05, ##*p* < 0.01, ###*p* < 0.001 versus Flag‐Vector with TGF‐*β*1 or H/R.

Previous study reported that SARS‐CoV N protein can interact with Smad3 and potentiate Smad3‐mdiated transcriptional responses of TGF‐*β*.^[^ ^8^ ^]^ Based on the high homology (90%) of the amino acid sequences and fewer mutations over time among coronavirus N proteins,^[^ ^37^ ^]^ we examined whether SARS‐CoV‐2 N protein can also interact with Smad3 in human embryonic kidney (HEK293T) cells by cotransfecting both Flag‐SARS‐CoV‐2 N (Flag‐N) and HA‐Smad3 expressing plasmids. Results in **Figure** **6**A,B showed that Flag‐N protein was able to interact with Smad3 to form a complex as determined by co‐immunoprecipitation (Co‐IP). Further studies in HEK293T, human TECs (HK‐2), and mouse TECs (mTEC) also revealed that overexpression of Flag‐N but not Flag‐VC was capable of promoting TGF‐*β*1 or hypoxia/reoxygenation (H/R)‐induced Smad3 signaling as demonstrated by enhancing TGF‐*β*1‐induced Smad3 phosphorylation, Smad3 nuclear translocation (Figure 6C–H), and Smad3 promoter luciferase activities (Figure 6I) in HEK293T cells. Further studies in HK‐2 cells also showed that overexpression of SARS‐CoV‐2 N largely enhanced H/R and TGF‐*β*1 induced p21‐dependent cell cycle arrest at the G1 phase (**Figure** **7**A–E), thereby blocking TEC from G1‐phase to the S‐phase cell cycle transition with few BrdU+ cells (Figures S4 and S5, Supporting Information).

**Figure 8:**
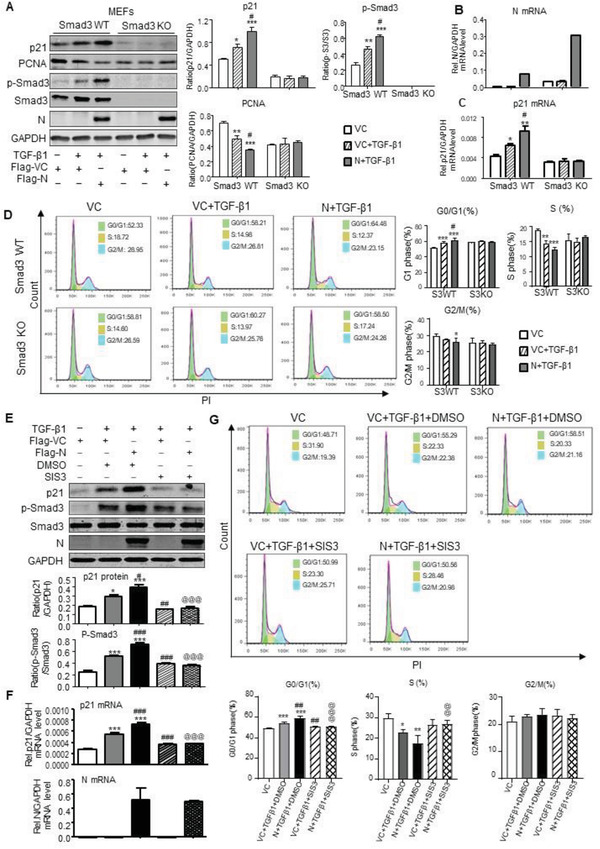
Overexpression of SARS‐CoV‐2 N protein induces cell cycle arrest at the G1 phase via a Smad3‐dependent mechanism in vitro. A–D) Smad3 KO/WT mouse embryonic fibroblasts (MEFs) with or without overexpressing SARS‐CoV‐2 N were treated with TGF‐*β*1 (5 ng mL^−1^) for 6 h for examining SARS‐CoV‐2 N expression, Smad3 signaling, and cell cycle progression by western blotting (A), real‐time PCR (B, C), and flow cytometry (D). Note that MEFs lacking Smad3 are protected against SARS‐CoV‐2 N‐induced G1 cell cycle arrest but promoted G1‐S progression in presence or absence of TGF‐*β*1. E–G) HK‐2 with or without overexpressing SARS‐CoV‐2 N were pretreated with SIS3 (10 µm) for 1 h and then TGF‐*β*1 (5 ng mL^−1^) for 6 h for examining SARS‐CoV‐2 N expression, Smad3 signaling, and cell cycle progression by western blotting (E), real‐time PCR (F), and flow cytometry (G). Results show that pretreatment with SIS3 blocks Smad3 signaling and inhibits SARS‐CoV‐2 N‐induced G1 cell cycle arrest but promotes the G1‐S progression in HK‐2 TECs in response TGF‐*β*1. Data was expressed as means ± SEM of three independent experiments. *p < 0.05, **p < 0.01, ***p < 0.001 versus Flag‐Vector; #*p* < 0.05, ##*p* < 0.01, ###*p* < 0.001 versus Flag‐Vector+TGF‐*β*1+DMSO; @@@ *p* < 0.001 versus Flag‐N+TGF‐*β*1+DMSO.

To further confirm the regulatory role of Smad3 in SARS‐CoV‐2 N‐induced cell death, we cultured Flag‐N‐overexpressing mouse embryonic fibroblasts (MEFs) with or without Smad3 gene KO. As shown in **Figure** **8**, western blot, real‐time PCR, and flow cytometry detected that compared with the Smad3 WT MEFs, although MEFs lacking Smad3 expressed high levels of SARS‐CoV‐2 N mRNA, they were protected from SARS‐CoV‐2 N‐induced cell cycle arrest at the G1 phase by blunting upregulation of p21 and cell cycle arrest at the G0/G1‐phase (Figure 8A–C), thereby largely promoting the MEFs entering into the S‐phase of cell cycle under high TGF‐*β*1 conditions (Figure 8D). These in vitro results confirmed the in vivo findings as shown in Figure 5 that Smad3 is a key mediator in SARS‐CoV‐2 N protein‐induced cell death via the p21‐dependent G1 cell cycle arrest mechanism.

### 2.5 Targeting Smad3 Inhibits SARS‐CoV‐2 N Protein‐Induced AKI

**Figure 9:**
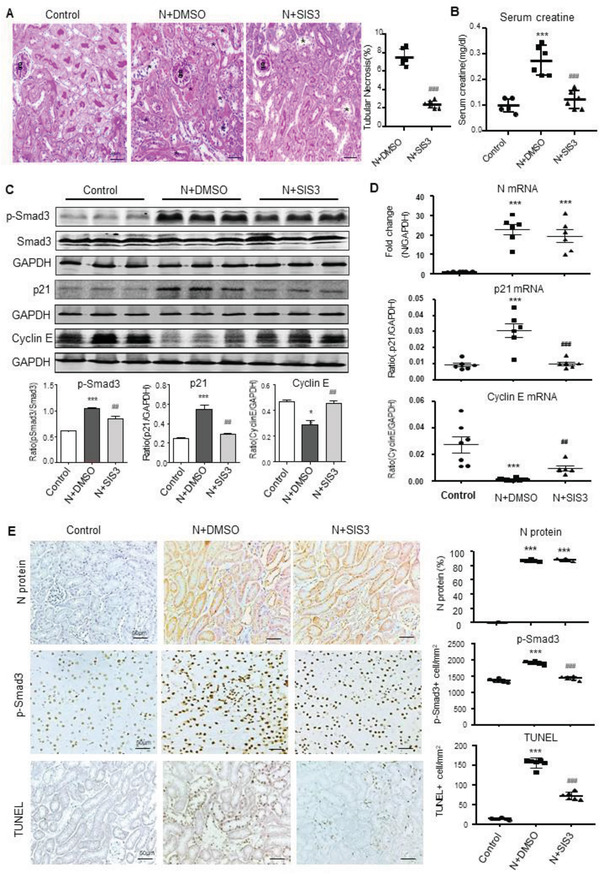
Treatment with a Smad3 inhibitor SIS3 inhibits SARS‐CoV‐2 N protein‐induced AKI in mice. A) PAS staining and semiquantitative analysis of tubular necrosis. B) Serum creatinine. C) Western blotting for activation of Smad3 (p‐Smad3) and expression of p21 and cyclin E protein. D) Real‐time PCR for mRNA levels of SARS‐CoV‐2 N, p21, and cyclin E expression. E) Immunohistochemistry for detecting SARS‐CoV‐2 N protein expression, activation of Smad3 signaling (p‐Smad3 nuclear translocation), and cell apoptosis by TUNEL‐labeling. Each dot represents one mouse and data are expressed as the mean ± SEM for groups of six mice. *p < 0.05, ***p < 0.001 versus control mice; ##*p* < 0.01, ###*p* < 0.001 versus SARS‐CoV‐2 N plasmid with DMSO (N+DMSO) group. g, glomerulus; scale bars = 50 µm .

We next tested our hypothesis that targeting Smad3 may be a novel therapy for SARS‐CoV‐2 N protein‐induced AKI, which was examined in SARS‐CoV‐2 N‐induced AKI mice by a daily intraperitoneal (i.p.) injection with a Smad3 inhibitor SIS3. We first determined an optimal therapeutic dosage of SIS3 for treatment of SARS‐CoV‐2 N‐induced AKI in mice by i.p. injection of SIS3 at dosages of 2.5, 5, and 10 mg kg^−1^ body weight daily from day 0 before kidney‐specifically SARS‐CoV‐2 N gene transfer until being sacrificed on day 2 (the peak time of AKI). Compared to DMSO control‐treated mice, although treatment with SIS3 did not alter the expression of SARS‐CoV‐2 mRNA and protein (Figure S6A,C, Supporting Information), it did significantly attenuate tubular necrosis and elevated serum levels of creatinine in a dose‐dependent manner, showing 5 mg kg^−1^ as the best therapeutic dose of SIS3 on AKI (Figure S6, Supporting Information). Because all of three dosages of SIS3 did not cause systemic and other organ toxicities as determined by LDH, ALT, and AST (Figure S7, Supporting Information), therefore, SIS3 at 5 mg/kg/day was selected as an optimal dosage for treatment of SARS‐CoV‐2 N‐induced AKI. As shown in Figure 9, treatment with SIS3 at 5 mg kg^−1^ body weight largely protects against SARS‐CoV‐2 N protein‐induced AKI by inhibiting tubular necrosis (**Figure** **9**A) and an increase in serum creatinine (Figure 9B). This therapeutic effect on AKI was associated with inhibition of Smad3 phosphorylation and its nuclear translocation without altering the expression of SARS‐CoV‐2 N mRNA and protein expression (Figure 9C–E). Further studies also found that treatment with SIS3 was able to protect kidneys from SARS‐CoV‐2 N‐induced cell death through the G1 cell cycle arrest by suppressing p21 while increasing cyclin E expression (Figure 9C,D), thereby inhibiting TEC apoptosis by largely reducing TUNEL‐positive cells (Figure 9E).

To further confirm the therapeutic mechanism of SIS3 in SARS‐CoV‐2 N protein‐induced TEC death via the cell cycle arrest at the G1 phase, we treated SARS‐CoV‐2 N‐overexpressing HK‐2 cells with a Smad3 inhibitor SIS3 in the presence of TGF‐*β*1. As shown in Figure 8E–G, western blot, real‐time PCR, and flow cytometry revealed that treatment with SIS3 was able to substantially block SARS‐CoV‐2 N protein‐induced Smad3 phosphorylation and G1 cell cycle arrest by suppressing p21 expression and the G0/G1 subpopulation while promoting the S‐phase cell cycle progression. BrdU labeling assay also confirmed this notion that addition of SIS3 was able to reverse the suppressive effect of SARS‐CoV‐2 N on the S‐phase cell cycle progression by largely increasing BrdU‐expressing HK‐2 cells (Figure S8, Supporting Information).

## 3 Discussion

In this study, we reported a novel finding that SARS‐CoV‐2 N protein is pathogenic and can induce AKI in normal mice and cause severe AKI under the ischemic stress condition. This was supported by the findings that ultrasound‐microbubble‐mediated kidney‐specifically overexpressing SARS‐CoV‐2 N protein was capable of dose‐dependently inducing AKI in normal mice by causing tubular necrosis and elevated serum levels of creatinine, which was further exacerbated in IRI‐mice under ischemic stress conditions. These experimental observations are consistent with the clinical notion that AKI is common in patients with severe COVID‐19 infection, which becomes worse with high mortality in those with critically ill and underlying ischemic and inflammatory stress conditions.^[^ ^34^ ^]^

It is well known that high levels of ACE2 expressing in renal TECs are responsible for the SARS‐CoV‐2 entry into the TECs via the S protein‐ACE2 interaction.^[^ ^38^ ^]^ This is supported by the evidence that SARS‐CoV‐2 S/N proteins and viruses‐like particles are detectable in the TECs of AKI patients who died from SARS‐CoV‐2 infection.^[^ ^14^, ^15^, ^16^, ^17^, ^39^ ^]^ Thus, the kidney, particularly TECs, is a target of COVID‐19, and AKI is common in critically ill patients with SARS‐CoV‐2 infection.^[^ ^38^, ^40^ ^]^ It is highly possible that after COVID‐19 infection, SARS‐CoV‐2 can enter into the TECs via the S‐ACE2 axis. The intracellular release of the N protein may trigger the TEC death, resulting in the development of AKI. This is supported by the finding that urinary SARS‐CoV‐2 and SARS‐CoV‐2 N protein can be detected in COVID‐19 patients with AKI and correlate with the AKI severity and mortality.^[^ ^41^, ^42^ ^]^ These clinical observations suggest the pathogenic role for SARS‐CoV‐2 N in AKI. Findings from the present study confirmed that this clinical notion unraveled the new role for SARS‐CoV‐2 N protein in the pathogenesis of AKI.

Consistent with the previous finding that SARS‐CoV‐associated coronavirus nucleocapsid protein can interact with Smad3 and specifically potentiates Smad3‐mediated transcriptional responses of TGF‐*β*, such as the activation of plasminogen activator inhibitor‐1 (PAI‐1),^[^ ^8^ ^]^ we also found that SARS‐CoV‐2 N protein can interact with Smad3 to form a complex to promote Smad3 signaling in response to TGF‐*β*1 by enhancing its phosphorylation and nuclear translocation. This may be associated with the high homology (90%) of the amino acid sequences among coronavirus N proteins (34). Importantly, we also uncovered that Smad3 is a key mediator responsible for SARS‐CoV‐2 N‐induced TEC death during the development of AKI. Indeed, TGF‐*β* is increased significantly in COVID‐19 patients.^[^ ^43^ ^]^ It is also well known that activation of TGF‐*β* signaling in TECs can induce AKI,^[^ ^44^ ^]^ which is blocked by deleting TGF‐*β*RII and Smad3 (26, 27). Results from this study provided new evidence for an essential role for Smad3 in SARS‐CoV‐2 N‐induced AKI as mice null for Smad3 were able to protect against SARS‐CoV‐2 N‐induced AKI. Thus, SARS‐CoV‐2 N protein can induce AKI via a Smad3‐dependent mechanism.

We further dissected the downstream mechanism through which Smad3 mediates SARS‐CoV‐2 N‐induced AKI and found that SARS‐CoV‐2 N induced AKI via the Smad3‐dependent G1 cell cycle arrest mechanism. It has been well defined that Smad3 can bind the promoters of many CKIs, such as p15, p16, p21, and p27.^[^ ^28^, ^30^, ^31^, ^32^ ^]^ We have also reported that Smad3 can directly bind to the CKD inhibitor p21/p27 to cause TEC death, resulting in the development of AKI under ischemic and inflammatory stress conditions.^[^ ^30^, ^33^ ^]^ Because the p21 protein can function to inhibit the cell cycle progression by arresting cell at the G1 phase through negatively regulating Cyclin E,^[^ ^30^, ^45^ ^]^ overexpression of SARS‐CoV‐2 N may activate Smad3 to trigger the TEC death pathway by upregulating p21 while suppressing cyclin E, thereby resulting in TEC arrest at the G0/G1 phase while preventing cells entering into the S phase. This was further supported by the findings that mice and cells null for Smad3 could protect against SARS‐CoV‐2 N protein‐induced cell death by largely blunting the p21‐dependent G1 cell cycle arrest pathway while promoting the G1‐S cell cycle progression and TEC proliferation in vivo and in vitro. Thus, Smad3 may mediate SARS‐CoV‐2 N protein‐induced TEC death during the development of AKI via a G1 cell cycle arrest mechanism.

Most significantly, we also found that targeting Smad3 with a pharmacological inhibitor SIS3, was able to inhibit SARS‐CoV‐2 N‐induced AKI. We found that treatment with SIS3 dose‐dependently inhibited SARS‐CoV‐2 N‐induced AKI in mice with altering the expressing levels of SARS‐CoV‐2 N in both mRNA and protein levels. Mechanistically, treatment with SIS3 was able to inactivate Smad3 signaling specifically and effectively and thus blocked p21‐dependent G1 cell cycle arrest and inhibited SARS‐CoV‐2 N‐induced TEC death and AKI. These findings suggest that targeting Smad3 may be a specific therapeutic strategy for SARS‐CoV‐2‐associated AKI.

It should be mentioned that TGF‐*β* is also a crucial mediator in tissue fibrosis.^[^ ^46^ ^]^ It is possible that a massive increase in local active TGF‐*β* in the lungs in response to COVID‐19 infection may promote lung inflammation and fibrosis and thus anti‐TGF‐*β* treatment for COVID‐19 has been proposed.^[^ ^47^ ^]^ A clinical trial of anti‐TGF‐*β* treatment with the OT‐101 (an antisense against TGF‐*β*2) has been reported to inhibit SARS‐CoV and SARS‐CoV‐2 replication and to protect COVID‐19 patients from going into respiratory crisis, massive edema, and fibrosis.^[^ ^48^ ^]^ Besides pulmonary fibrosis which is known to be a major complication in COVID‐19 patients,^[^ ^49^ ^]^ liver fibrosis is also reported in patients with COVID‐19.^[^ ^50^ ^]^ Furthermore, a study using multiorgan proteomic landscape of COVID‐19 autopsies indicates that many fibrosis‐associated proteins are detectable in the lung, liver, heart, and kidney, suggesting a multiorgan profibrotic state in the COVID‐19 patients.^[^ ^51^ ^]^ As Smad3 is also a key mediator of tissue fibrosis,^[^ ^46^ ^]^ it is likely that the prolong, persistent, or repeated COVID‐19 infection may be able to continuously activate Smad3, resulting in AKI‐to‐CKD progression. Therefore, targeting Smad3 may also be a novel therapeutic strategy for COVID‐19‐related organ fibrosis, which may be warranted for further studies.

There are several limitations of the present study. First, the present study was based on the kidney‐specific SARS‐CoV‐2 N expressing plasmid transfection, rather than the live SARS‐CoV‐2 virus infection. Thus, the results from the present study may not be able to directly apply to COVID‐19 patients with AKI clinically. Second, SARS‐CoV‐2 N protein‐induced AKI may involve multiple mechanisms and may not limit to the Smad3‐dependent G1 cell cycle arrest mechanism. Multiple factors such as direct virus infection,^[^ ^14^, ^52^ ^]^ cytokine storm,^[^ ^53^, ^54^ ^]^ and hypoxia^[^ ^55^ ^]^ are also involved in the pathogenesis of COVID‐19‐associated AKI. Previous studies demonstrated that SARS‐CoV‐2 N protein can be found in kidney tubules and are colocalized with the ACE2 protein.^[^ ^14^, ^52^ ^]^ The expression of hypoxic damage‐associated molecules, including DP2 and prostaglandin D synthase, were co‐expressed in renal tubular cells with SARS‐CoV‐2 N protein antigens, suggesting SARS‐CoV‐2 virus can directly initiate hypoxic damage in infected kidney tubules.^[^ ^14^ ^]^ The inflammatory response triggered by SARS‐CoV‐2 infection can recruit and activate monocytes/macrophages and dendritic cells, and induces the secretion of cytokines, resulting in the cytokine storm, hypoxia‐associated stress, and the development of AKI, which is more profound in critically ill COVID‐19 patients.^[^ ^53^, ^54^, ^56^, ^57^, ^58^ ^]^ Thus, further studies on the role and mechanisms of inflammation‐related pathways in the pathogenesis of COVID 19‐associated AKI are warranted.

In summary, SARS‐CoV‐2 N protein can induce AKI and exacerbate AKI under ischemic and inflammatory stress conditions. SARS‐CoV‐2 N protein induces AKI by causing TEC death via the Smad3‐dependent G1 cell cycle arrest mechanism. Thus, targeting Smad3 may be an effectively therapeutic potential for COVID‐19‐associated AKI.

## 4 Experimental Section

### Preparation of SARS‐CoV‐2 N Protein Expressing Plasmid

Mammalian expression plasmids for pcDNA3.1(+)‐Flag‐N were constructed and synthesized by GeneScript (Nanjing, China) and the GenBank accession number was MW617760.1. The expression plasmid of Smad3 was cloned into C‐terminal HA‐tagged pCAGGS vector with the cDNA template purchased from WZ Biosciences (China). The primers used in this study were as follows:

Flag‐N: F: GCGGATCCATGTCTGATAATGGACCCCA;
R:GCTCTAGATTAGGCCTGAGTTGAGTCAG;
HA‐Smad3: F: CATCATTTTGGCAAAGAATTCATGTCGTCCATCCTGCCTTTC;
R: TGCATCGATGAGCTCGAATTCAGACACACTGGAACAGCGGATG.

PcDNA3.1(+)‐Empty‐Vector and pcDNA3.1(+)‐Flag‐N were purified by EndoFree Plasmid Giga Kits (12391, QIAGEN, Germantown, MD, GER).

### Mouse Models of AKI Induced by Kidney‐Specifically Overexpressing SARS‐CoV‐2 N Protein

The dose‐dependent effect of SARS‐CoV‐2 N on induction of AKI in normal mice was first determined. The SARS‐CoV‐2 N protein‐expressing plasmid (N) or empty control vector (VC) at 100, 200, 300 µg/mouse was mixed with SonoVue microbubbles (Bracco Diagnostics) at the 1:1 ratio in volume. Total of 400 µL mixture was injected into normal C57BL/6 mice (male, 8–10 weeks) via tail vein, followed immediately by placing the ultrasound probe (Therasonic 450 Dual Frequency Ultrasound) on the back of mouse opposite the bilateral kidneys and treated with ultrasound at a plus‐wave output (2 W cm^−2^) for a total of 15 min with the 30‐s interval to transfect the N gene specifically into the mouse kidneys as previously described.^[^ ^35^, ^36^ ^]^ The mice (*n* = 3) were sacrificed at day 3 for selecting the optimal dose for induction of AKI without systemic toxicities. In addition, a group of three normal mice without any treatment were used as normal control. As shown in Figures S1 and S2, Supporting Information, it was found that a dose of 200 µg/mouse was able to induce AKI by increasing serum levels of creatinine and the severity of tubular necrosis without detectable systemic toxicities as determined by serum LDH assay, AST assay, and ALT assay. Thus, SARS‐CoV‐2 N‐expressing plasmid at 200 µg/mouse was selected as an optimal dosage for the study.

The authors then determined the time‐dependent effect of SARS‐CoV‐2 N protein on AKI by intravenously injecting normal mice with an optimal dose (200 µg/mouse) of SARS‐CoV‐2 N protein (or control vector)‐expressing plasmid/SonoVue mixture via tail vein, followed immediately by ultrasound exposure as described above. Groups of three mice were sacrificed daily over the next 5 days for examining the peak time of SARS‐Cov‐2 N protein expression. In addition, a group of three mice were used as normal control. As shown in Figure S3, Supporting Information, SARS‐CoV‐2 N protein expression peaked at day 2, which was accompanied with severe AKI as determined by serum creatine and tubular necrosis. Thus, time on day 2 after SARS‐CoV‐2 N gene transfer was selected for the study. Thus, a mouse model of AKI was induced in normal C57BL/6 mice by kidney‐specifically transfecting SARS‐CoV‐2 N gene or control vector at 200 µg/mouse and groups of six mice were sacrificed at day 2 for examining the SARS‐CoV‐2 N mRNA and protein expression, TGF‐*β*/Smad3 signaling, cell death, and the severity of AKI.

To examine the pathogenic role of SARS‐CoV‐2 N protein in AKI under ischemic conditions mimicking critically ill COVID‐19 patients clinically, an ischemic mouse model of AKI was induced by a well‐established IRI in mice after 2 days of kidney‐specifically SARS‐CoV‐2 N gene or control empty vector transfer (200 µg/mouse) as described above. Briefly, after anesthesia by the i.p. injection of ketamine/xylazine mixture, a mouse model of ischemic AKI was induced in SARS‐CoV‐2 N‐overexpressing mice on day 2 by bilaterally clamping renal arteries with vascular clip for 30 min as described previously.^[^ ^30^, ^33^, ^45^ ^]^ Control mice received the sham‐operation procedures without clamping the renal arteries. All mice after surgery received 5% glucose and NaCl intraperitoneally for volume supply and buprenorphine intramuscular for analgesia. Groups of six mice were sacrificed at 24 and 72 h after IRI.

As SARS‐CoV N protein could interact with Smad3 to transcriptionally regulate the PAI activation,^[^ ^8^ ^]^ the authors then investigated the mechanism though which SARS‐CoV‐2 N protein induces AKI in Smad3 KO and WT mice. Briefly, Smad3 KO C57BL/6 mice were kindly provided by Dr. C. Deng and the mouse genotypes were determined by genotyping the tail DNA as previously described.^[^ ^59^ ^]^ A mouse AKI model was induced in Smad3 KO or WT mice by kidney‐specifically overexpressing SARS‐CoV‐2 N protein mediated by a noninvasive ultrasound‐microbubble technique as described above. Groups of six mice were sacrificed at day 2 after the N gene transfection for examining the SARS‐CoV‐2 N mRNA and protein expression, TGF‐*β*/Smad3 signaling, cell death, and the severity of AKI.

To determine cell proliferating activities through the S phase, mice were i.p. injected with BrdU (ab‐142567, Abcam, Cambridge, UK) at a dose of 50 mg kg^−1^ 2 h before sacrificed. All animal experimental protocols were approved by the Animal Ethics Experimentation Committee at the Chinese University of Hong Kong.

### Treatment of SARS‐CoV‐2 N Protein‐Induced AKI with a Smad3 Inhibitor SIS3

The authors next developed a therapeutic approach for SARS‐CoV‐2 N‐induced AKI by targeting Smad3 with a pharmacological Smad3 inhibitor SIS3 (Sigma‐Aldrich, St. Louis, MO) as previously described.^[^ ^30^ ^]^ The dose‐dependent effect of SIS3 on SARS‐CoV‐2 N protein‐induced AKI was first determined by treating C57BL/6 mice with SIS3 at dosages of 2.5, 5, and 10 mg/kg/day intraperitoneally from day 0 before ultrasound‐microbubble‐mediated SARS‐CoV‐2 N gene transfer. All mice were sacrificed at day 2 after the N gene transfer for determining the optimal dose with the best therapeutic effect on AKI without systemic toxicities. As shown in Figures S6 and S7, Supporting Information, SIS3 at a dose of 5 mg/kg/day was selected as an optimal dosage for the treatment study. Briefly, groups of 6 C57BL/6 mice (male, 8–10 weeks) were treated with i.p. injection of SIS3 at 5 mg/kg/day from day 0 before the SARS‐CoV‐2 N gene transfer until being sacrificed on day 2 (the peak time of AKI) for determining the therapeutic effect of SIS3 on SARS‐CoV‐2 N mRNA and protein expression, TGF‐*β*/Smad3 signaling, cell death, and the severity of AKI. The animal experimental protocol was approved by the Animal Ethics Experimentation Committee at the The Chinese University of Hong Kong.

### Cell Lines and Cell Cultures

The mTECs were a gift from Dr. Jeffrey B. Kopp (NIH). Smad3 WT or KO MEFs were isolated from Smad3 WT and Smad3 KO mice as previously described.^[^ ^60^ ^]^ Briefly, the embryos were decapitated and eviscerated, then triturated in 0.25% trypsin/1 nm EDTA for 30 min at 37 °C and suspended and cultured in Dulbecco's modified Eagle's medium (DMEM) (Gibco, Grand Island, NY, USA) with 10% FBS, 100 U mL^−1^ penicillin, and 100 µg mL^−1^ streptomycin sulfate. Characterized MEFs from Smad3 WT or KO mice were used for studying the regulatory mechanisms of Smad3 in SARS‐CoV‐2 N‐induced cell death in vitro. Human embryonic kidney (HEK293T) and human tubular epithelial (HK‐2) cells were purchased from the American Type Culture Collection (ATCC) (Manassas, VA, USA). HEK293T cells and MEFs with or without overexpressing SARS‐CoV‐2 N were cultured in DMEM supplemented with 10% FBS, 100 U mL^−1^ penicillin, and 100 µg mL^−1^ streptomycin sulfate. HK‐2 and mTEC cells with or without overexpressing SARS‐CoV‐2 N were cultured in DMEM/F12 medium (Gibco) supplemented with 10% FBS, 100 U mL^−1^ penicillin, and 100 µg mL^−1^ streptomycin sulfate. Cells were stimulated with or without TGF‐*β*1 (5 ng mL^−1^, Peprotech 100‐21C) for different time points. For H/R injury, HK‐2 cells were cultured in the AnaeroPack system with oxygen less than 0.1% (Mitsubishi Gas Chemical, Japan) at 37 °C for 12 h, then returned to normal conditions with 95% air and 5% CO2 for 6 h.

To study the inhibitory effect of SIS3 on SARS‐CoV‐2 N protein‐induced cell cycle progression, HK‐2 cells overexpressing SARS‐CoV‐2 N protein were pretreated with a pharmacological inhibitor of Smad3, SIS3 (10 µm) (Sigma, S0447), 1 h prior to addition of TGF‐*β*1 (5 ng mL^−1^). To determine the S‐phase cell cycle progression, 10 µm BrdU (ab‐142567, Abcam) were added into the cultured HK‐2 cells 2h before being harvested. All cell cultures were maintained in an incubator at 37 °C in a humidified atmosphere of 5% CO_2_ and at least three‐independent experiments were performed in each study.

### Renal Function and Histology

Blood was collected by cardiac puncture when sacrificed under anesthesia for measuring serum creatinine using the quantitative enzymatic method of the Creatinine LiquiColor Test (Endpoint) (Stanbio Laboratory, Texas, USA) according to the manufacturer's protocol. Kidney tissues were fixed with Histochoice Tissue Fixation MB (AMRESCO, VWR Life Science, PA, USA). PAS staining (Sigma) was performed in paraffin sections (3 µm). A total of 500 cortical tubules were examined and the numbers of tubules with necrosis was counted and expressed as percentage.

### Measurement of Serum Lactate Dehydrogenase, Alanine Aminotransferase, and Aspartate Aminotransferase Levels

To detect the potential toxicities of SARS‐CoV‐2 N gene transfer and SIS3 treatment systemically to irrelevant organs, AST assay kit and ALT assay kit (Najing Jiancheng Bioengineering Institute, Nanjing, China) were used for the AST and ALT detection, and the LDH assay kit (Najing Jiancheng Bioengineering Institute, Nanjing, China) was used for LDH release according to the manufacturer's protocol.

### Immunohistochemistry

Immunohistochemistry was performed on paraffin kidney sections (3 µm) using a microwave‐based antigen retrieval technique.^[^ ^61^ ^]^ The antibody used in this study including sheep polyclonal antibody to BrdU (ab‐1893, Abcam), rabbit polyclonal antibody to PCNA (sc‐7907, Santa Cruz Biotechnology, Dallas, TX), p‐Smad2/3 (600‐401‐919, ROCKLAND, Limerick, PA, USA), and SARS‐CoV‐2 N protein (ab273167, Abcam). After incubated with the primary antibody overnight at 4 °C, the sections were washed and incubated with rabbit anti‐Goat immunoglobulins/horseradish peroxidase (HRP) (0049, DAKOP, Santa Clara, CA, USA) or EnVision+ System‐HRP Labeled Polymer anti‐Rabbit (4003, DAKOK) at room temperature for 1 h, then color was developed with 3,3'‐diaminobenzidine (DAB) (045‐22833, Bio connect life sciences, Huissen, HOL) and viewed under a Leica CRT6000 Light Microscope. The positive cells were counted in ten random areas of kidney sections under the power field of microscope and expected as cells/mm^2^.

### Opal Multiplex Immunofluorescence

Paraffin kidney sections (3 µm) were treated with a microwave‐based antigen retrieval technique^[^ ^61^ ^]^ and incubated with primary antibodies, including rabbit polyclonal antibody to SARS‐CoV‐2 N protein (ab‐273167, Abcam), p‐Smad2/3 (600‐401‐919, ROCKLAND), and BrdU (ab‐1893, Abcam) at 4 °C for overnight. Sections were incubated with EnVision+ System‐HRP Labeled Polymer Anti‐Rabbit (4003, DAKOK) at room temperature for 1 h; then the fluorescence was developed using the Alex Fluor 488 Tyramide Reagent (B40953, Invitrogen, Carlsbad, CA, USA) or Alex Fluor 555 Tyramide Reagent (B40955, Invitrogen) according to the manufacturer's protocol. The nuclei were counterstained with Hoechst 33342, Trihydrochloride, Trihydrate (H1399, Invitrogen). All slides were mounted with medium and then analyzed with Leica CRT6000 fluorescence microscope.

### Cell Apoptosis Assay

Apoptosis in the kidney section was detected with the TUNEL Assay Kit‐HRP‐DAB (ab‐206386, Abcam) according to the manufacturer's protocol. Results were imaged with a Leica CRT6000 Light Microscope. The positive cells were counted in ten random areas of kidney sections under the power field of microscope and expected as cells/mm^2^.

### Cell Cycle Analysis

To examine the effect of N protein on cell cycle in S3WT or S3KO MEF cells and HK2 cells, all cells were fixed by 70% alcohol in PBS at 4 °C overnight. After washing with PBS, cells were then incubated with 20 µg mL^−1^ propidium iodide (ab14083, Abcam) and 1% RNaseA in PBS in dark at 37 °C for 30 min. After being extensively washed with PBS, cells were resuspended in PBS and analyzed by FACS Calibur flow cytometry (BD Biosciences, San Jose, CA).

### Nuclear and Cytoplasmic Extraction

The Thermo Scientific NE‐PER Nuclear and Cytoplasmic Extraction Kit (78835, Invitrogen) was used for efficient cell lysis and extraction of cytoplasmic and nuclear protein fractions and then analyzed by western blot with the antibodies. Antibody against Flag (F3165) was purchased from Sigma (St. Louis, MO, USA). Antibody against p‐Smad3 (ab52903) was purchased from Abcam (Abcam). Antibody against GAPDH was purchased from Millipore (Burlington, MA, USA). Antibody against Lamin A/C was obtained from Cell Signaling Technology (4777S, Beverly, MA, USA).

### Western Blot Analysis

HK‐2 cell lysates were prepared by RIPA lysis buffer (P0013B, Beyotime). Protein concentration was determined by Bradford assay (Bio‐Rad, Hercules, CA, USA). The cell lysates (30 µg) were electrophoresed in an 8–12% SDS‐PAGE gel and transferred to a Nitrocellulose Membrane (Bio‐Rad). Nitrocellulose membrane was blocked with 5% skim milk in Tris‐buffered saline with 1% Tween 20 (TBS‐T) before being incubated with target antibody. The signals were visualized by the Odyssey Infrared Imaging System (San Diego, CA) and quantitatively analyzed by normalizing to GAPDH using the Image J software (NIH, Bethesda, MD).

### Co‐Immunoprecipitation Assays

The Dynabeads Protein G Immunoprecipitation Kit (10007D, Invitrogen) was used for immunoprecipitation assays. Cell lysates were prepared by lysing cells with buffer (50 mm Tris‐HCl, pH 7.5, 300 mm NaCl, 1% Triton‐X, 5 mm EDTA, and 10% glycerol). Lysates were immunoprecipitated with anti‐HA antibody (H6908, Sigma) or anti‐Flag antibody (F3165, Sigma) with Dynabeads Protein G. The Dynabeads‐Ab‐Ag complex was washed three times and then eluted for western blot analysis.

### Luciferase Reporter Assay

In a 24‐well plate, HEK293T cells were transfected with a mixture of 200 ng of 12× (CAGA) luciferase reporter (firefly luciferase) and 20 ng of pRL‐TK (Renilla luciferase plasmid), together with empty vector plasmid, HA‐Smad3 plasmid, or Flag‐N plasmid for 24 h. Luciferase activity was measured by using a Dual‐Luciferase Reporter Assay System kit (Promega, San Luis Obispo, CA) according to the manufacturer's protocol. Data represent relative firefly luciferase activity, normalized to Renilla luciferase activity.

### Real‐Time PCR

Total RNA was extracted with TRIzol reagent (Invitrogen) following the manufacturer's instructions. Real‐time quantitative‐PCR was performed using QuantStudio 7 Flex and SYBR Green Supermix (Bio‐Rad). Real‐time PCR primers were designed by Primer Premier 5.0 and their sequences were as follows:

Human p21 forward, 5′‐AGTCAGTTCCTTGTGGAGCC‐3′,
Human p21 reverse, 5′‐CGCAGAAACACCTGTGAACG‐3′.
Mouse p21 forward, 5′‐AGGCATATCTAGGCACTTGC‐3′,
Mouse p21 reverse, 5′‐CCACACACCATAGAATGCTC‐3′.
SARS‐CoV‐2 N forward, 5′‐GAAATGCACCCCGCATTACG‐3′,
SARS‐CoV‐2 N reverse, 5′‐GTGAGAGCGGTGAACCAAGA‐3′.
Mouse Cyclin E forward, 5′‐GCAGGCGAGGATGAGAGCAGT‐3′,
Mouse Cyclin E reverse, 5′‐CCCGGAGCAAGCGCCATCTGTA‐3′.
Human GAPDH forward, 5′‐GAAGGTGAAGGTCGGAGTC‐3′,
Human GAPDH reverse, 5′‐GAAGATGGTGATGGGATTTC‐3′.
Mouse GAPDH forward, 5′‐GGTGAAGGTCGGTGTGAACG‐3′,
Mouse GAPDH reverse, 5′‐CTCGCTCCTGGAAGATGGTG‐3′.

### Statistical Analyses

Each experiment was repeated at least three times. All values were expressed as the mean ± SEM. Statistical analysis were performed with the *t*‐test for two groups or one‐way ANOVA from GraphPad Prism5 (GraphPad Software, San Diego, CA, USA) for multiple groups. *p* values less than 0.05 were considered statistically significant.

## Conflict of Interest

The authors declare no conflict of interest.

## Supporting information

Supporting Information

## Data Availability

The data that support the findings of this study are available from the corresponding author upon reasonable request.
