# Supplementary material for: SARS‐CoV‐2 N Protein Induces Acute Kidney Injury via Smad3‐Dependent G1 Cell Cycle Arrest Mechanism
Source: Adv Sci (Weinh). 2021 Nov 23;9(3):2103248. doi: 10.1002/advs.202103248 (PMC8787402; doi:10.1002/advs.202103248)
Supplement: Supplementary file 1 — Supporting Information [file ADVS-9-2103248-s001.pdf]

ADVANCED SCIENCE
Open Access

Supporting Information

for *Adv. Sci.*, DOI: 10.1002/advs.202103248

SARS-CoV-2 N protein induces acute kidney injury via Smad3-dependent G1 cell cycle arrest mechanism

*Wenbiao Wang[1,2,4,6,#], Junzhe Chen[1,3,#], Dingwen Hu[5], Pan Pan[2], Liying Liang[1], Wenjing Wu[1,4], Ying Tang[3], Xiao R Huang[1,4], Xueqing Yu[4,**], Jianguo Wu[2,5,**], Hui Y Lan[1,6,*]*

Supplementary Information

SARS-CoV-2 N protein induces acute kidney injury via Smad3-dependent G1 cell cycle arrest mechanism

Wenbiao Wang[1,2,4,6,#], Junzhe Chen[1,3,#], Dingwen Hu[5], Pan Pan[2], Liying Liang[1], Wenjing Wu[1,4], Ying Tang[3], Xiao R. Huang[1,4], Xueqing Yu[4,**], Jianguo Wu[2,5,**], Hui Y. Lan[1,6*].

[1] Departments of Medicine & Therapeutics, Li Ka Shing Institute of Health Sciences, and Lui Che Woo Institute of Innovative Medicine, The Chinese University of Hong Kong, Hong Kong, China.

[2] Guangdong Provincial Key Laboratory of Virology, Institute of Medical Microbiology, Jinan University, Guangzhou 510632, China.

[3] Department of Nephrology, The Third Affiliated hospital, Southern Medical University, Guangzhou, China.

[4] Guangdong-Hong Kong Joint Laboratory for Immunological and Genetic Kidney Disease, Guangdong Academy of Medical Science, Guangdong Provincial People's Hospital, Guangzhou, China.

[5] State Key Laboratory of Virology, College of Life Sciences, Wuhan University, Wuhan 430072, China.

[6] The Chinese University of Hong Kong-Guangdong Academy of Sciences/Guangdong Provincial People's Hospital Joint Research Laboratory on Immunological and Genetic Kidney Diseases, The Chinese University of Hong Kong, Hong Kong, China.

[#]Wenbiao Wang, Junzhe Chen contributed equally to this study

***Correspondence**: Hui-Yao Lan, Departments of Medicine & Therapeutics, Li Ka Shing Institute of Health Sciences, The Chinese University of Hong Kong, Hong Kong, China. E-mail: hylan@cuhk.edu.hk.

****Co-correspondence**: Jianguo Wu, jwu898@jnu.edu.cn; Xueqing Yu, yuxq@mail.sysu.edu.cn

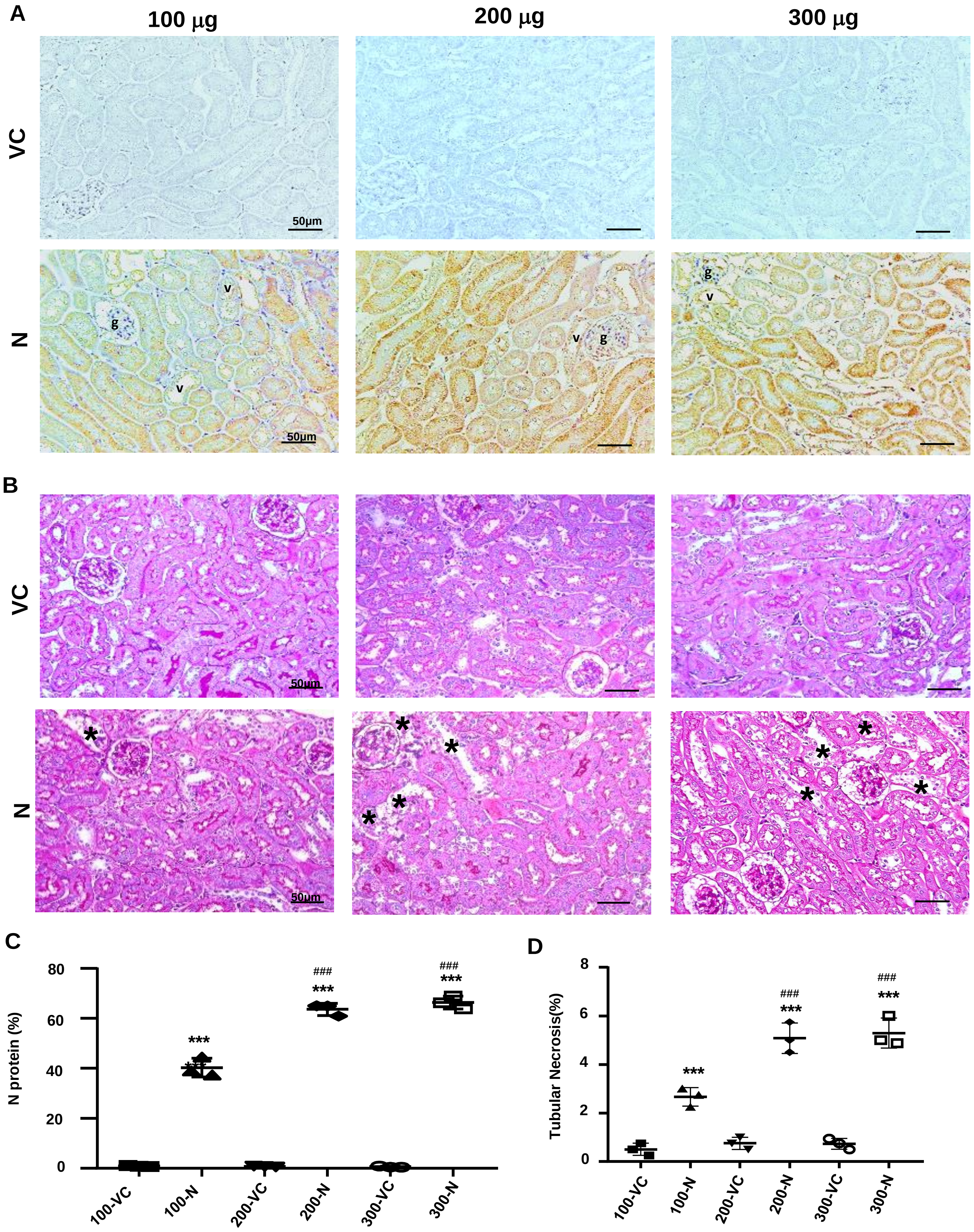

Figure S1. Ultrasound-microbubble-meditated kidney-specifically SARS-CoV-2 N protein (N) expression induces tubular necrosis in a dose-dependent manner. (A) N protein immunostaining; **(B)** PAS-staining; **(C)** Semi-quantitative analysis of N protein; **(D)** Semi-quantitative analysis of tubular necrosis (*). Note that SARS-CoV-2 N protein plasmid transfer results in overexpression of N protein and tubular necrosis in a dose-dependent manner, peaking at 200 μg/mouse. Each dot represents one mouse and data are expressed as the mean ± SEM for groups of 3 mice. ***p<0.001 versus vector control (VC); ###p<0.001 versus SARS-CoV-2 N-expressing plasmid at 100 μg/mouse (100-N). g, glomerulus; v, vascular cells. Scale bar=50 μm.

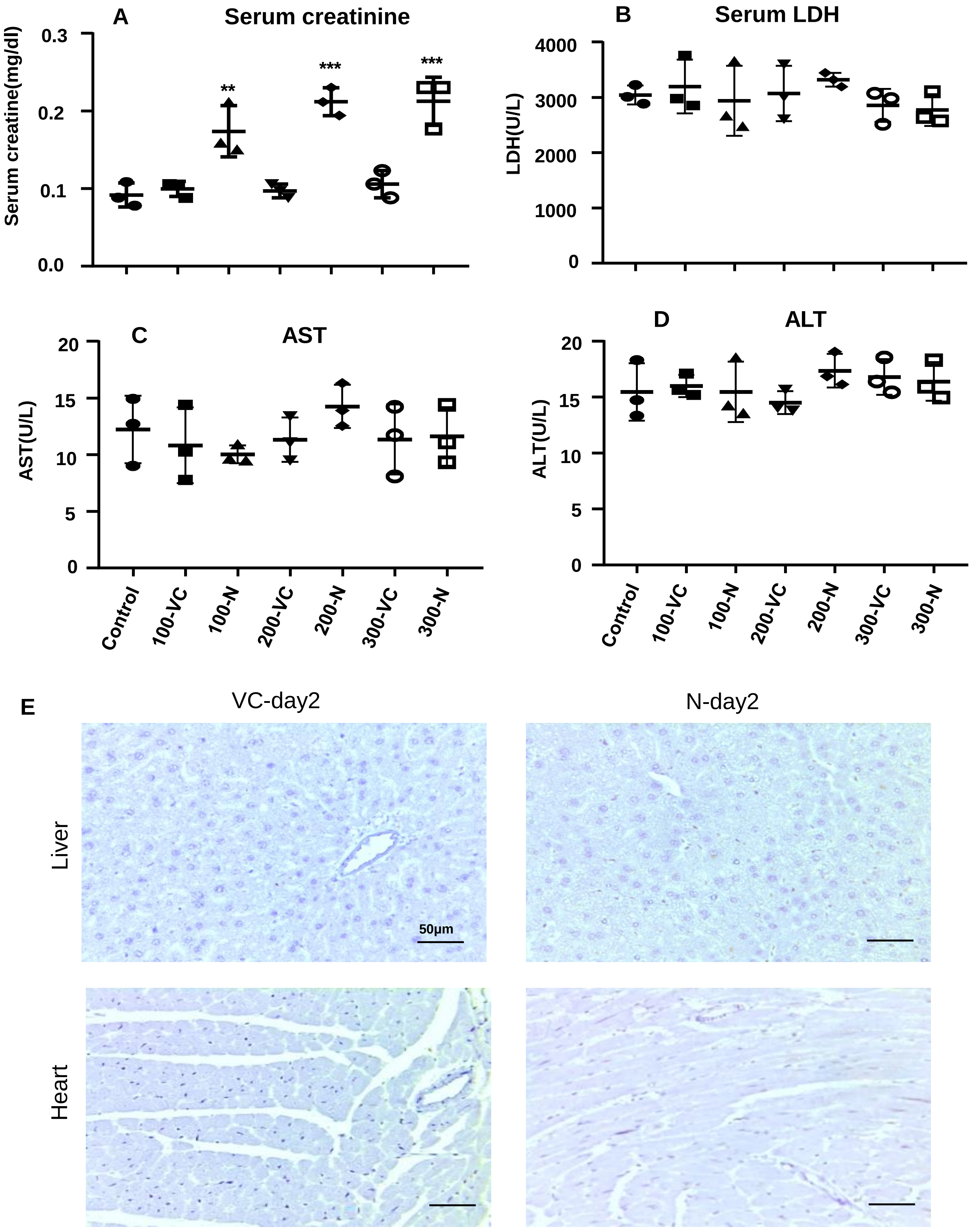

Figure S2. Ultrasound-microbubble-meditated kidney-specifically SARS-CoV-2 N protein (N) expression results in a significant increase in serum creatine in a dose-dependent manner without systemic toxicity. (A) Serum creatine; **(B)** serum LDH; **(C)** AST; **(D**) ALT; **(E)** immunohistochemical staining shows that expression of SARS-CoV-2 N protein is not detectable in both liver and hear tissues after ultrasound-microbubble-mediated kidney-specifically SARS-CoV-2 N transfection. Each dot represents one mouse and data are expressed as the mean ± SEM for groups of 3 mice.**p<0.01, ***p<0.001 versus vector control (VC).

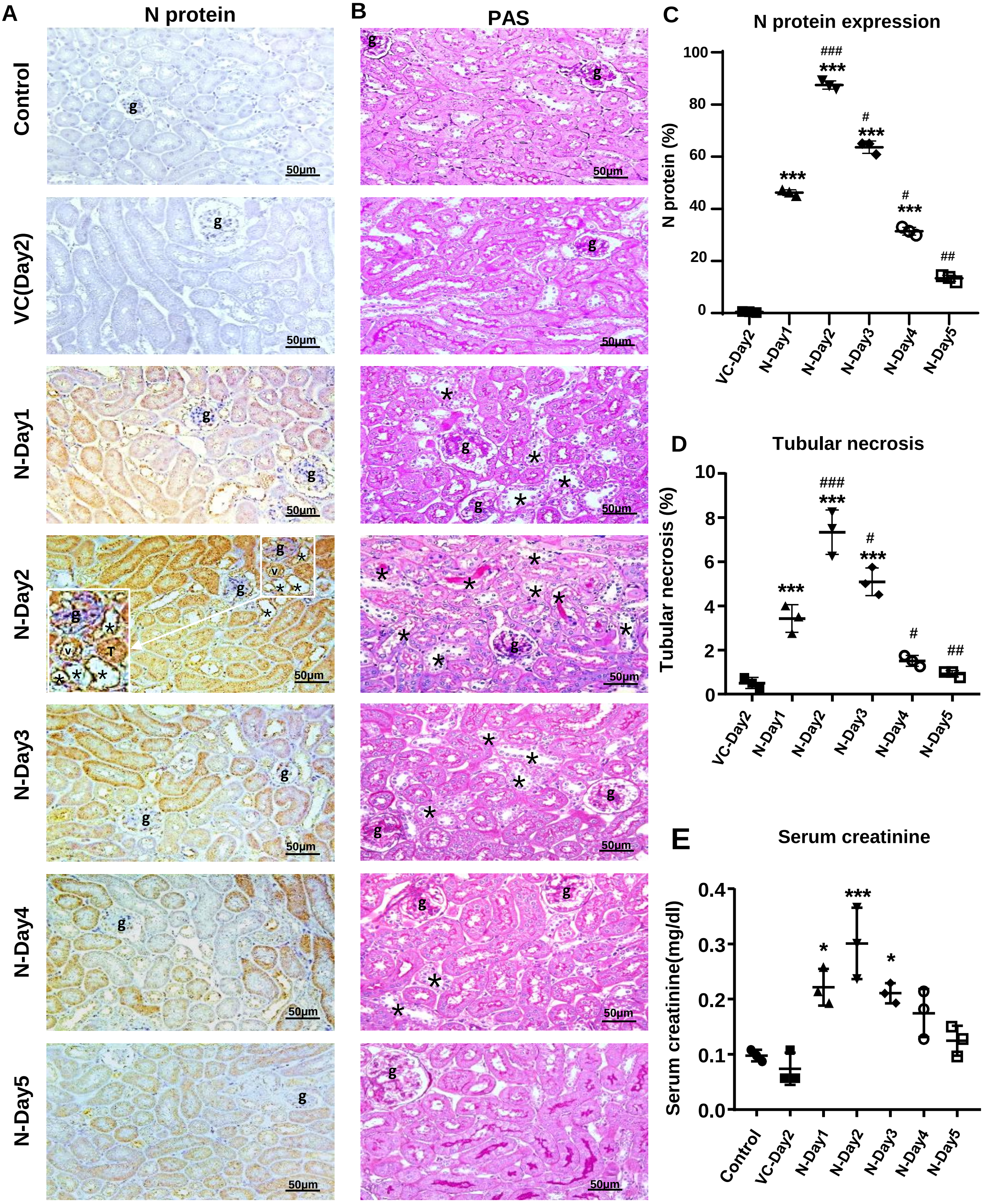

Figure S3. Kidney-specifically overexpressing SARS-CoV-2 N protein (N) causes AKI in a time-dependent manner, peaking at day 2 after the N gene transfer. (A) N protein immunostaining; **(B)** PAS-staining; **(C)** Semi-quantitative analysis of N protein; **(D)** Semi-quantitative analysis of tubular necrosis (*); **(E)** Serum levels of creatinine. Note that kidney-specifically SARS-CoV-2 N-expressing plasmid transfer results in overexpression of N protein primarily in tubular cells with tubular necrosis (*) as well as in glomerular (g) and vascular (v) cells (see inserted picture at day 2) in a time-dependent manner, peaking at day 2. Each dot represents one mouse and data are expressed as the mean ± SEM for groups of 3 mice. *$p<0.05$, ***$p<0.001$ versus vector control (VC); #$p<0.05$, ##$p<0.01$, ###$p<0.001$ versus SARS-CoV-2 N-expressing plasmid at day 1 (N-day 1). g, glomerulus. Scale bar=50 μm.

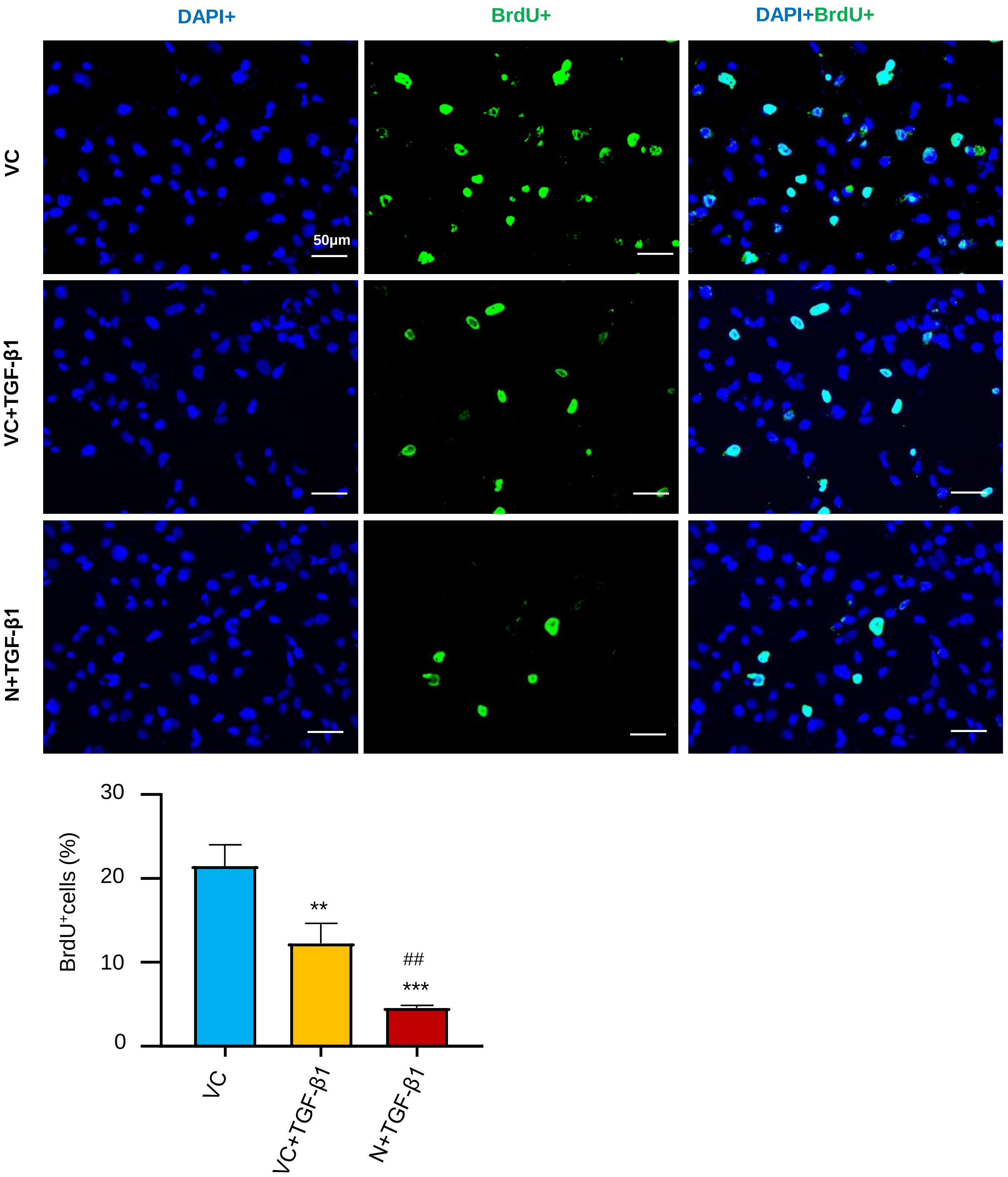

Figure S4. Immunofluorescence detects that overexpressing SARS-CoV-2 N protein (N) largely promotes TGF-β1-induced inhibition of S-phase cell cycle in human TECs (HK-2) in vitro. Note that under normal conditions, HK-2 cells are highly proliferative as demonstrated by many cells entering into the S-phase of cell cycle, which is inhibited by addition of TGF-β1 (5ng/ml) and becomes worsen by co-overexpressing SARS-CoV-2 N protein. Each bar represents the mean ± SEM for 3 independent experiments.**p<0.01, ***p<0.001 versus vector control (VC); ## p<0.001 versus VC + TGF-β1. Scale bar=50 μm.

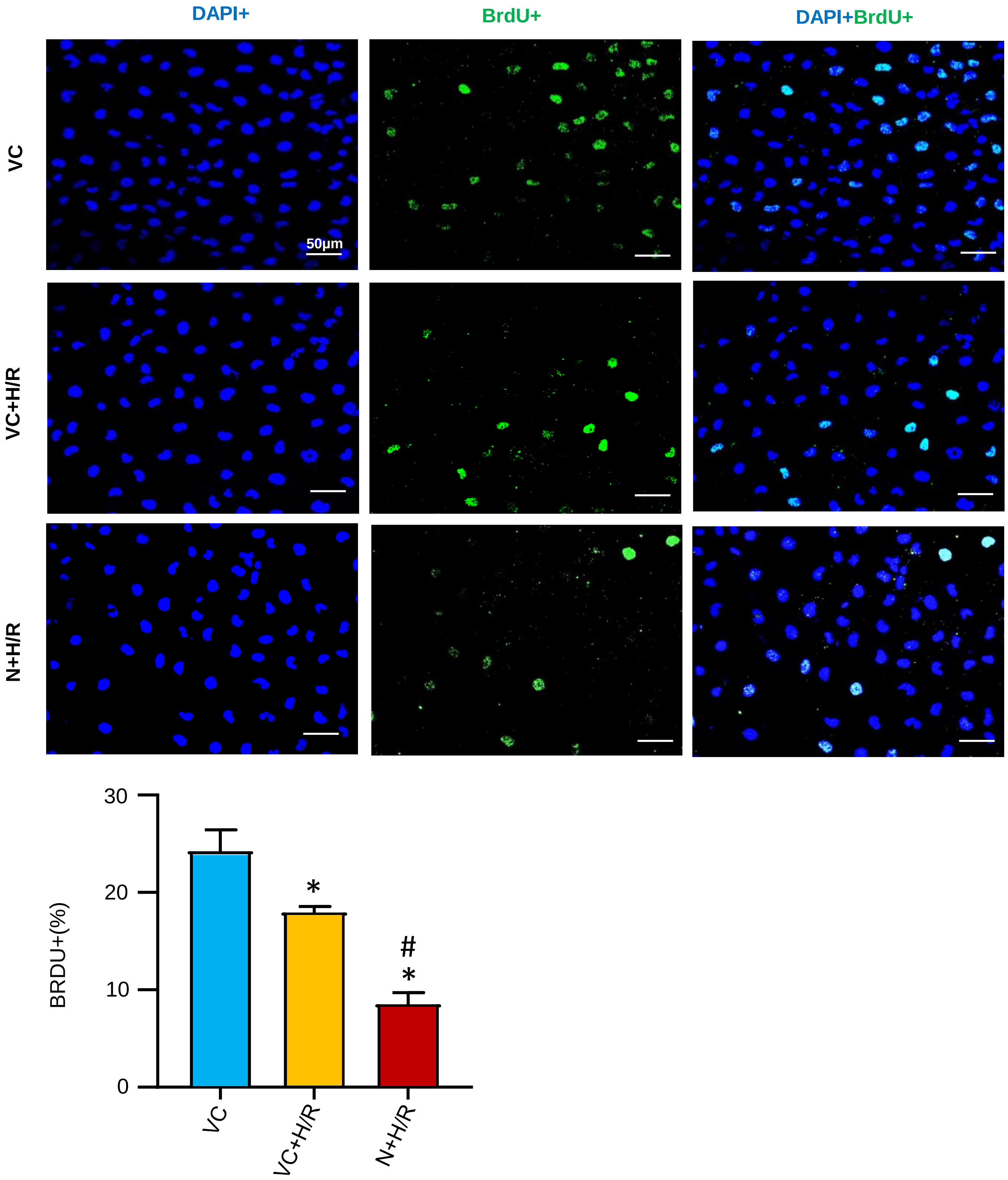

Figure S5. Immunofluorescence detects that overexpressing SARS-CoV-2 N protein (N) largely promotes hypoxia/reoxygenation-induced inhibition of S-phase cell cycle in human TECs (HK-2) in vitro.

Note that under normal conditions, HK-2 cells are highly proliferative as demonstrated by many cells entering into the S-phase of cell cycle, which is inhibited by hypoxia/reoxygenation injury and becomes worsen by overexpressing SARS-CoV-2 N protein. Each bar represents the mean ± SEM for 3 independent experiments. .*p<0.05 versus vector control (VC); # p<0.005 versus VC +H/R. Scale bar=50 μ m.

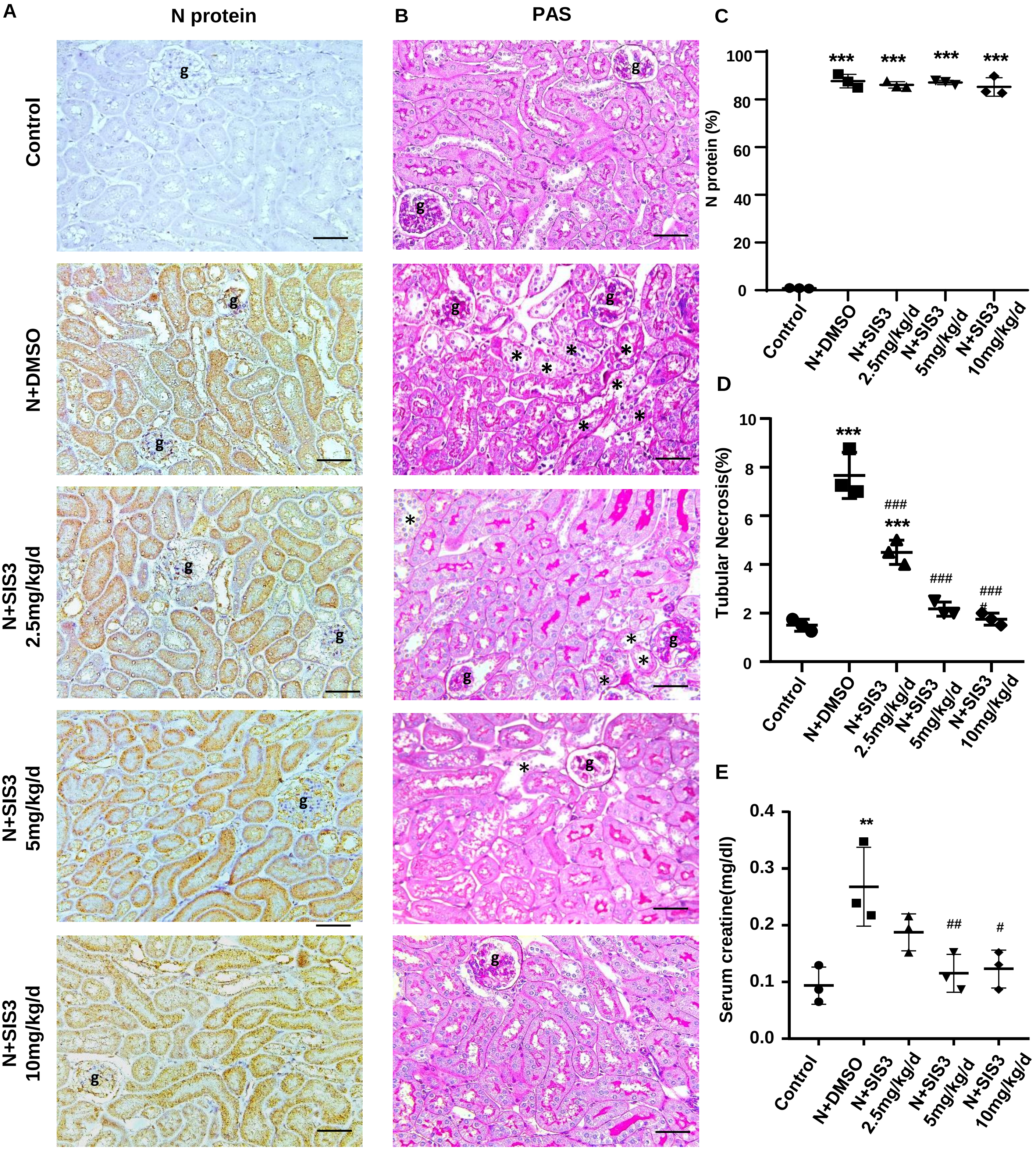

Figure S6. Treatment with SIS3 dose-dependently inhibits SARS-CoV-2 N protein-induced AKI without altering expression of N protein in the kidneys. (A) SARS-CoV-2 N protein immunostaining; **(B)** PAS-staining for detection of tubular necrosis (*); **(C)** Semi-quantitation of the N protein expression in the kidney; **(D)** Semi-quantitation of renal tubular necrosis; **(E)** Serum levels of creatinine. Each dot represents one mouse and data are expressed as the mean ± SEM for groups of 3 mice. **$p<0.01$, ***$p<0.001$ versus vector control; #$p<0.05$, ##$p<0.01$, ###$p<0.001$ versus overexpression of SARS-CoV-2 N + DMSO-control treatment (N+DMSO). g, glomerulus. Scale bar=50 μm.

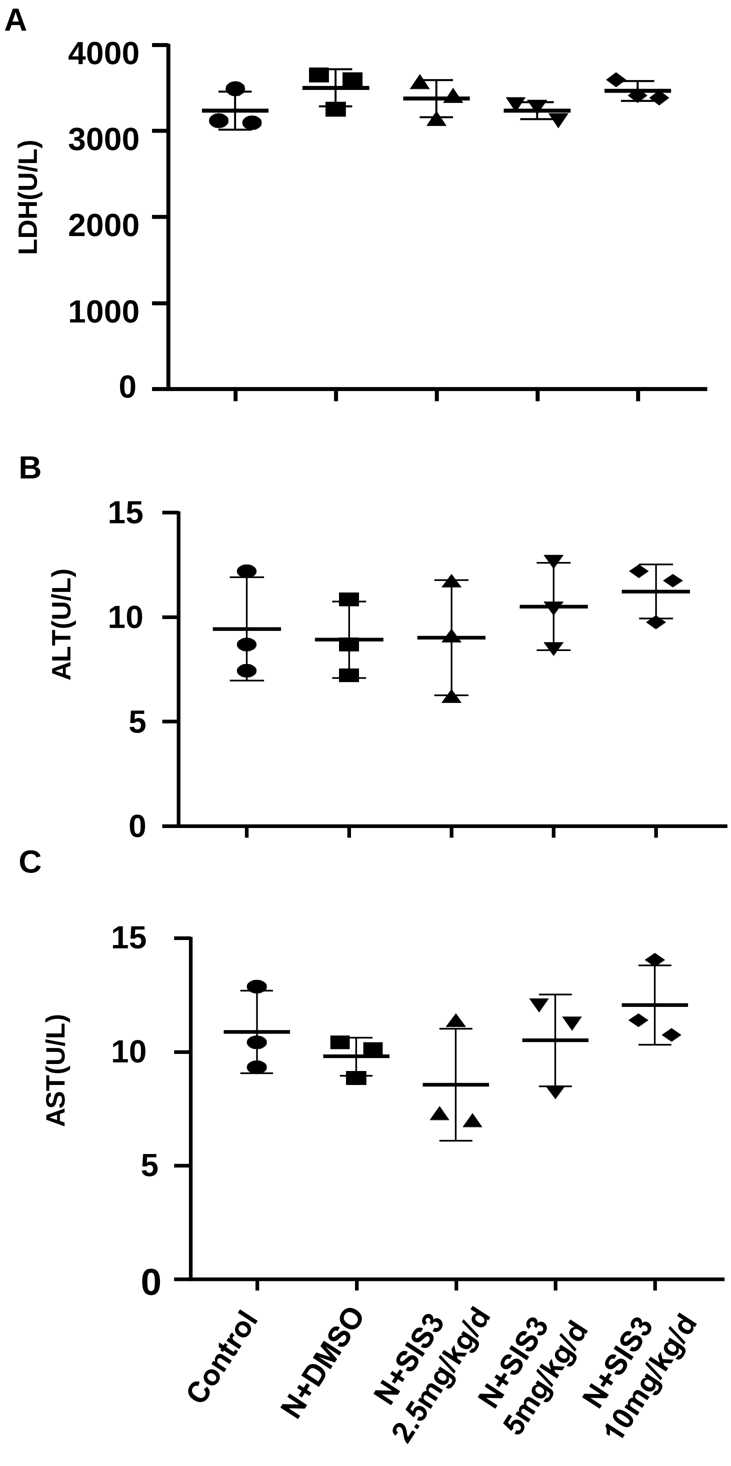

Figure S7. Systemic toxicity of SIS3 in mice with SARS-CoV-2 N protein-induced AKI. (A) Serum levels of LDH release; (**B)** Serum levels of ALT; **(C)** Serum levels of AST. Each dot represents one mouse and data are expressed as the mean ± SEM for groups of 3 mice.

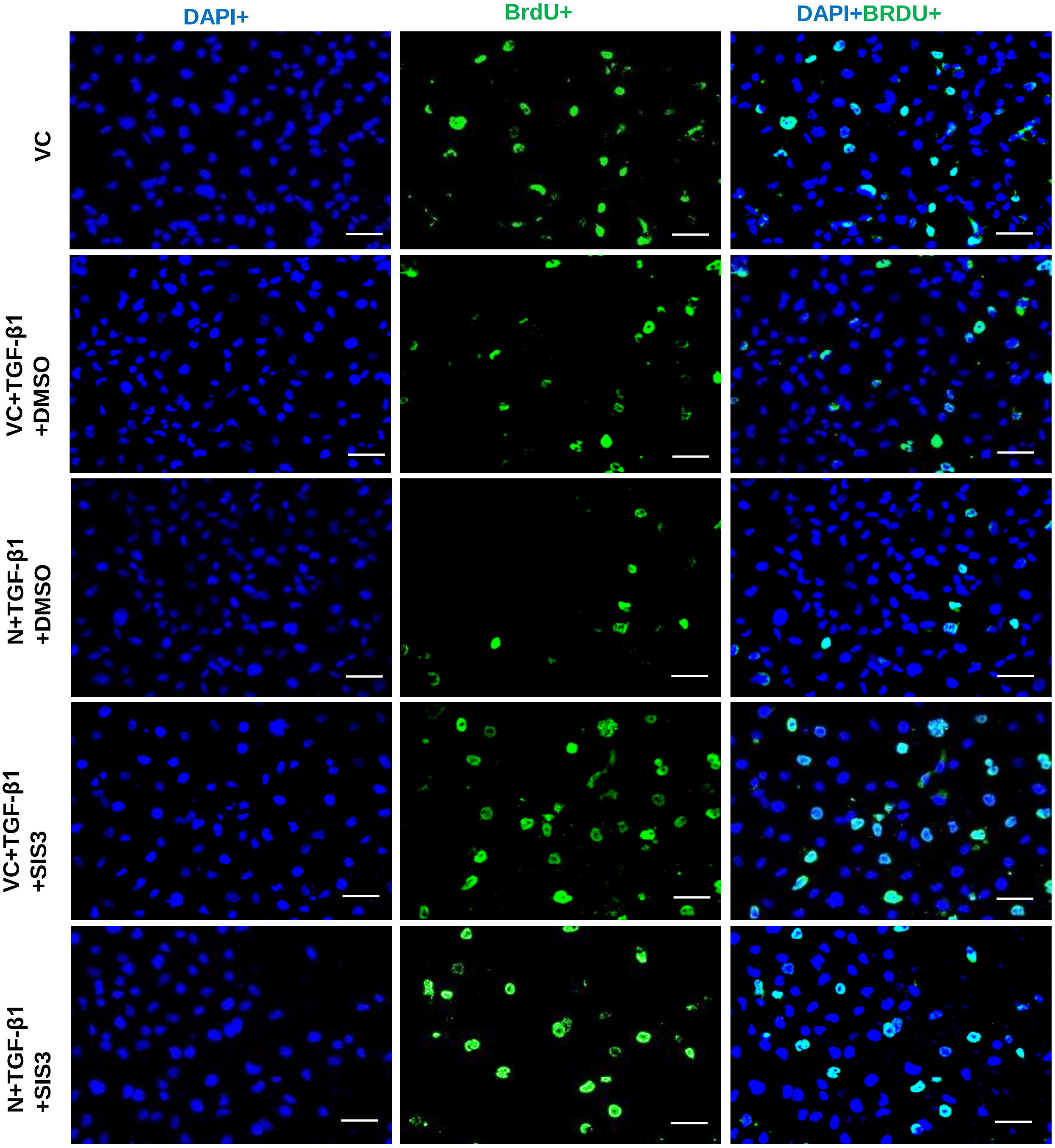

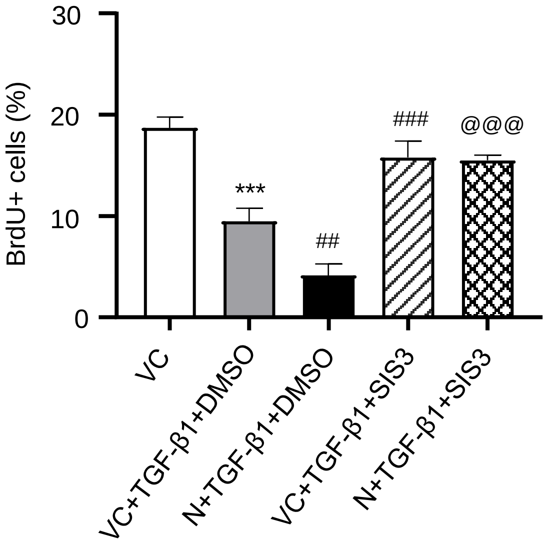

Figure S8.Blockade of Smad3 reverses SARS-CoV-2 N-induced inhibition of cell cycle progression at the S-phase in HK-2 cells in vitro. Immunofluorescence detects that overexpression of SARS-CoV-2 N protein (N) can severely impair TEC proliferation by blocking the S-phase cell cycle progression , which is virtually blocked with a Smad3 inhibitor SIS3. Note that SIS3 treatment also block TGF-β1-induced inhibition of S-phase cell cycle in HK-2 cells. Each bar represents the mean ± SEM for groups of 3 independent experiments. *$p<0.05$, ***$p<0.001$ versus vector control (VC); ##$p<0.01$, ###$p<0.001$ versus VC+TGF-β1+DMSO; @@@ $p<0.001$ versus N+TGF-β1+DMSO. Scale bar=50 μm.
